# How Much Does Fusion Design Matter? Partition, Seed, and Cluster-Aware Evaluation of Hybrid LSTM–GAT Traffic Anomaly Detectors

**DOI:** 10.3390/s26216744

**Published:** 2026-10-24

**Authors:** Ayhan Gültekin, Kerem Kartal, Ahmet Yusuf Şimşek, Rümeysa Yeşilova, Ahmet Sayar

**Affiliations:** 1 Student Affairs Department, Kocaeli University, 41001 Kocaeli, Türkiye; ayhan.gultekin@kocaeli.edu.tr; 2 Department of Software Engineering, Kocaeli University, 41001 Kocaeli, Türkiye; 210229019@kocaeli.edu.tr; 3 Department of Artificial Intelligence and Machine Learning, Kocaeli University, 41001 Kocaeli, Türkiye; 240121023@kocaeli.edu.tr (A.Y.Ş.); 250121044@kocaeli.edu.tr (R.Y.); 4 Department of Artificial Intelligence Engineering, Bahçeşehir University, 34353 Istanbul, Türkiye; 5 KOU Teknopark, Bidist, 41275 Kocaeli, Türkiye; 6 Department of Computer Engineering, Kocaeli University, 41001 Kocaeli, Türkiye

**Keywords:** traffic anomaly detection, spatio-temporal modeling, long short-term memory, graph attention networks, multimodal fusion, ablation study, grouped cross-validation, cluster bootstrap, permutation tests

## Abstract

Architectural assessments of traffic anomaly detectors can be sensitive to data partitioning, random training seeds, and dependence among overlapping windows. We evaluate nine encoder configurations, four fusion operators, and two graph constructions using physics-informed synthetic trajectories generated on an OpenStreetMap-derived road network. Each fold–seed threshold is selected on validation data and applied to its corresponding test set. Trip-level cluster bootstrap and permutation tests account for dependence among windows, while corrected resampled tests approximate the dependence induced by overlapping training sets. On a fixed partition of 14 test trips, no comparison establishes superiority after Holm correction. Across five-fold cross-validation with five seeds, mean F1 scores range from 0.8558 to 0.8839 across fusion operators, with gating and cross-attention producing occasional degenerate runs. Across 500 runs covering repeated grouped partitions and both graph constructions, no pairwise architectural comparison remains significant after correction. With run-specific thresholds, subtype analyses do not support the abnormal-dwell improvement that appears under pooled thresholds. Classical baselines remain competitive, but their apparent advantage in single-seed pooled tests is not corroborated by run-level inference. Overall, the results demonstrate that architectural conclusions are sensitive to threshold selection, the number of training seeds, and the choice of statistical unit of analysis.

## 1. Introduction

Intelligent Transportation Systems increasingly rely on spatio-temporal data to monitor traffic conditions and support operational decisions. Traffic anomalies range from unusual network-level states, such as abnormal speed or flow patterns, to events involving an individual vehicle or interaction. These problem settings differ in their observational units and input data, but both require detecting behavior that departs from expected temporal and spatial context. Recent traffic anomaly detectors use graph-based temporal models to represent dependencies among road-network nodes [1,2], while driving-video methods jointly model agent interactions and scene context [3].

This study focuses on a narrower setting: classifying fixed-length vehicle-trajectory windows using kinematic sequences and road-network context. A long short-term memory (LSTM) network can encode temporal changes in speed and acceleration, while a graph attention network (GAT) can represent relationships among connected road segments. Combining the two is plausible, but the best fusion mechanism is not self-evident: concatenation or weighted sums impose different constraints from sample-wise gating or cross-attention. Existing traffic anomaly studies demonstrate the value of spatio-temporal or interaction-aware representations, but they do not isolate these fusion choices in the trajectory-window classification task evaluated here. The study therefore asks whether encoder choice, fusion operator, or graph construction yields a reproducible performance advantage under a shared protocol.

Answering this question requires accounting for the structure of the evaluation data as well as model variability. Sliding windows overlap and are nested within trips, so treating every window as an independent test observation can understate uncertainty. Moreover, a single training seed or data partition cannot characterize variability in architectural rankings; in our implementation, repeated GPU runs with the same nominal configuration and seed also produce different scores. Accordingly, the evaluation groups data by trip, selects decision thresholds using validation data only, and reports results across multiple seeds and grouped partitions, with inference at the trip or run level as appropriate.

We investigate encoder pairing, fusion design, and graph construction in a hybrid LSTM–GAT detector, referred to as STANet (Spatio-Temporal Attention Network), which initially uses a sample-wise scalar modality gate. We compare this gate with alternative fusion operators under a common task, optimization budget, checkpoint-selection criterion, and validation-threshold policy. The experiments use physics-informed synthetic trajectories on an OpenStreetMap-derived road network, with replication on a second synthetic network; conclusions about performance therefore remain contingent on the simulator and require validation on field-collected trajectories. Each analysis reports the estimated quantities and number of training seeds.

The main contributions are:

- A controlled comparison of nine encoder configurations and four fusion operators, including task-specific adaptations of operators from the spatio-temporal graph convolutional network (STGCN), diffusion convolutional recurrent neural network (DCRNN), and Graph WaveNet;
- Corrected out-of-fold threshold handling, trip-level inference, and repeated grouped partitions with shared seeds, addressing distinct sources of evaluation uncertainty;
- Graph-construction and contextual-feature controls, together with gate diagnostics, which distinguish observed training failures from evidence of an architectural advantage;
- Rule-based and classical baselines, results for all anomaly subtypes, computational measurements, and sensitivity analyses of windowing, imputation, noise, prevalence, and independently generated synthetic datasets.

The remainder of the paper is organized as follows. Section 2 reviews related work on spatio-temporal anomaly detection, fusion strategies, and evaluation protocols. Section 3 describes the STANet architecture, dataset, preprocessing, and experimental protocol. Section 4 presents and discusses the results, including the architectural comparisons, robustness analyses, and threats to validity. Section 5 concludes the paper and outlines future work. Additional generation diagnostics, operator settings, and detailed sensitivity results are provided in the final sections.

## 2. Related Work

The literature most relevant to this study covers both anomaly detection models and the fusion strategies used to integrate temporal and spatial information.

### 2.1. Temporal, Spatial, and Spatio-Temporal Anomaly Models

Temporal models, including recurrent architectures such as LSTM networks, capture changes in velocity, acceleration, and short-term driving behavior. Such representations are relevant when anomalies are expressed primarily through kinematic deviations, but they may omit road context needed to interpret a maneuver. Graph-based models represent road connectivity, interactions between segments, and topological constraints. Combining these representations allows the contribution of sequential behavior and structural context to be evaluated within a common framework.

Recent studies increasingly bridge these two views through spatio-temporal learning. Ref. [4] combines Dynamic GAT and Informer components for generic multivariate time-series anomaly detection. Ref. [5] extends this direction through a multimodal deep-fusion framework with heterogeneous graph neural networks. Ref. [6] studies coupled speed–volume patterns in an urban traffic-forecasting setting. These studies motivate joint temporal and relational modeling across different tasks. Their application domains and prediction targets differ, however, so they do not directly establish the relative contribution of either branch in the present trajectory-classification task.

### 2.2. Fusion Strategies and Context Integration

A second research direction focuses on how information from temporal and spatial branches should be fused. Ref. [7] examines compact spatio-temporal feature fusion for network-traffic anomaly detection, whereas Ref. [8] uses a spatial–temporal attention design for lane-aware trajectory prediction. These designs motivate attention-based and feature-level integration, but they address different tasks and domains. Their findings therefore provide limited direct evidence about the relative performance of simpler fusion operators in the present setting.

The mechanism examined in this paper is a sample-wise scalar modality gate applied after the LSTM and GAT outputs have been projected into a shared 64-dimensional space. It produces one scalar per sample and broadcasts this value across the latent features, providing modality-level weighting distinct from feature, temporal, and graph-neighborhood attention. The cross-validated and repeated-partition analyses assess whether this modality-level distinction yields a measurable advantage, without interpreting nonsignificance as equivalence.

Transfer from simulated trajectories to real operating conditions remains an open question for these detectors. The present evaluation includes replication on a second synthetic road network; validation using field-collected trajectories remains a future direction.

### 2.3. Evaluation Protocols and Empirical Scope

Table 1 condenses the task context and evaluation practices of the selected studies and the present work. Their tasks and datasets differ, so reported performance values provide context rather than a common benchmark; detailed protocols and findings are retained in Appendix A Table A1. The comparison is illustrative rather than systematic. Among the five selected prior studies, two report five-run summaries, but none reports training seed identifiers or confidence intervals for between-model differences, and none combines repeated grouped partitions with shared training seeds.

The present evaluation identifies four sources of sensitivity. First, identical-seed replication of the LSTM–GAT configuration yields a mean absolute F1 difference of 0.0195 and a maximum of 0.0414. Second, expanding the primary CV from two to five seeds changes the fusion ranking and reveals degenerate gated and cross-attention runs; repeated-partition inference establishes neither superiority nor equivalence. Third, none of the sixteen fixed-partition comparisons is significant under trip-level F1 inference with within-family Holm correction; differences from window-level tests also involve a change in test statistic. Fourth, applying each run’s validation-selected threshold reduces the abnormal-dwell difference to +0.0074 F1, with a trip-bootstrap interval spanning zero. These findings show how replication, training variability, clustered observations, and threshold assignment qualify architectural conclusions in this synthetic task.

## 3. Materials and Methods

### 3.1. Problem Formulation

Urban traffic systems can be represented as spatio-temporal processes in which vehicle dynamics evolve within the structural constraints of a road network [9,10]. In this study, the traffic anomaly detection problem is formulated as a context-aware learning task that jointly models vehicle trajectory dynamics and road network topology [1]. The notation used in this formulation is summarized in Table 2.

#### 3.1.1. Road Network Representation

Following the graph-based road-network representations widely adopted in traffic anomaly detection [11,12], the road network is represented as a directed graph G=(V,E), where V is a set of *N* nodes (road segments) and E denotes the set of directed edges representing physical connectivity between segments [12]. Each node $v_{i}\in V$ has the structural feature vector $s_{i}$ defined by Equation (1):

(1) $$s_{i}=\left[type_{i},length_{i},deg_{i},context_{i}\right]$$

Equation (1) groups the descriptors by category; the implemented vector expands these categories into 17 dimensions. Here, $type_{i}$ denotes the road category and $length_{i}$ is the segment length, following the static road features adopted in Ref. [13]; $deg_{i}$ is the node degree, computed directly from the road-network graph; and $context_{i}$ represents additional intersection characteristics, such as intersection type, orientation, and connection angle, similar to the intersection-level attributes used in Refs. [13,14]. Spatial dependencies are encoded via an adjacency matrix *A* [15,16].

#### 3.1.2. Vehicle Trajectory Modeling

Vehicle movement is represented as a trajectory sequence $T=\{x_{1},x_{2},\ldots ,x_{\tau }\}$, where τ is the sequence length and each observation $x_{t}$ describes the kinematic state at time *t* according to Equation (2) [17]:

(2) $$x_{t}=\left[v_{t},a_{t},\theta_{t},id_{t}\right]$$

where $v_{t}$ is speed, $a_{t}$ is acceleration, $\theta_{t}$ is the heading angle, and $id_{t}$ is the identifier of the road segment where the vehicle is located.

#### 3.1.3. Spatio-Temporal Feature Representation

Spatio-temporal anomaly models integrate behavioral dynamics with structural context [18,19]. The learning task produces the joint embedding *z* defined by Equation (3):   

(3) $$z=f_{\mathrm{fusion}}\left(h_{temp},h_{spat}\right)$$

In the implementation, $f_{\mathrm{fusion}}$ operates on the projected embeddings in Equations (13) and (14). Here, $h_{temp}$ and $h_{spat}$ are defined in Equations (6) and (7), respectively. The function $f_{\mathrm{fusion}}\left(\cdot \right)$ denotes the fusion operator. Whereas prior studies typically adopt a fixed operator, such as weighted aggregation [20] or concatenation [21], it is treated in this study as an experimental variable rather than a fixed design choice.

#### 3.1.4. Traffic Anomaly Detection Objective

The detection task is formulated as binary classification, with anomaly probability defined by Equation (4):

(4) $$\hat{y}=\sigma \left(g\left(z\right)\right)$$

where g(·) is the classification head and $\hat{y}\in \left[0,1\right]$. The corresponding unweighted binary cross-entropy is defined by Equation (5):

(5) $$L=-\frac{1}{M}\sum_{i=1}^{M}\left[y_{i}\log\left({\hat{y}}_{i}\right)+\left(1-y_{i}\right)\log\left(1-{\hat{y}}_{i}\right)\right]$$

where $y_{i}\in \left\{0,1\right\}$ is the ground-truth label and *M* is the number of samples [22]. The loss in Equation (5) defines the general classification objective. To account for class imbalance in the training partition, the implemented objective uses the weighted binary cross-entropy in Equation (20).

#### 3.1.5. Problem Definition

Given a road network G, vehicle trajectory T, and road features S, the objective is to learn a mapping Φ:{T,G,S}→{0,1} [1,13].

### 3.2. Architecture Under Study

The model jointly encodes temporal vehicle dynamics and spatial road-network topology through parallel branches and combines them with a configurable fusion operator.

#### 3.2.1. Overall Architecture

**Figure 1:**
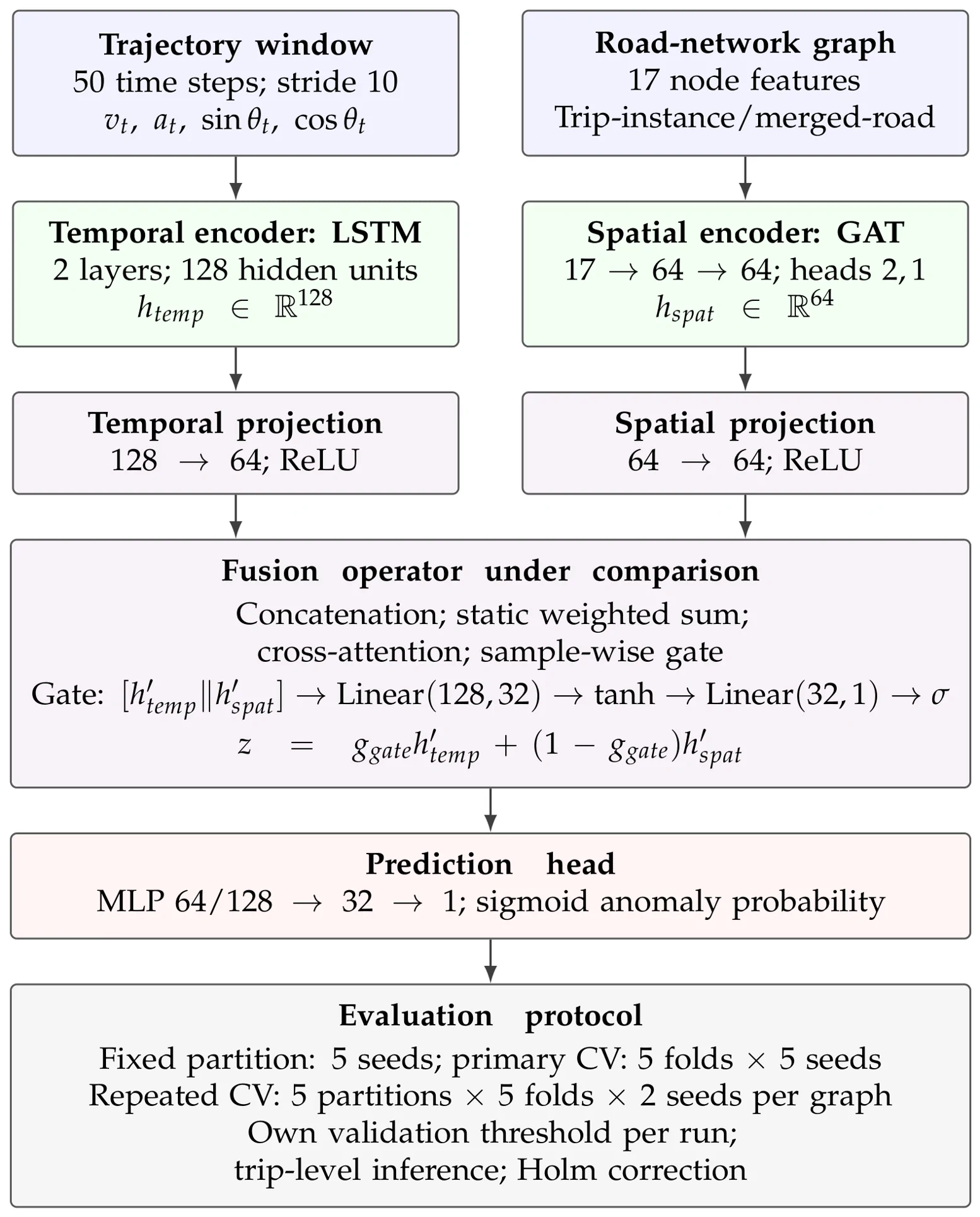
Hybrid LSTM–GAT architecture.

As illustrated in Figure 1, the framework consists of (i) a temporal module for sequence modeling, (ii) a spatial module for structural graph learning, and (iii) a fusion module that combines the two projected representations. The GAT neighborhood coefficients $\alpha_{ij}$ weight adjacent nodes, whereas the sample-wise scalar modality gate $g_{gate}$ weights the two branch representations. The fusion comparison holds both encoders fixed while varying the fusion operator.

Given a vehicle trajectory sequence T and the road network graph G, the model learns temporal and spatial embeddings through two parallel branches. The temporal branch employs an LSTM [23], and the spatial branch employs a GAT [24], following hybrid LSTM–GAT designs used in spatio-temporal traffic modeling [21]:

(6) $$\begin{array}{cc} h_{temp} & =f_{LSTM}\left(T;\Theta_{temp}\right) \end{array}$$

(7) $$\begin{array}{cc} h_{spat} & =f_{GAT}\left(G,A;\Theta_{spat}\right) \end{array}$$

where $\Theta_{temp}$ and $\Theta_{spat}$ denote the learnable parameter sets of the two branches. Unlike Ref. [21], where the LSTM outputs are passed to the GAT sequentially, the two embeddings here are computed independently and integrated through the fusion mapping defined by Equation (8):

(8) $$z=f_{\mathrm{fusion}}\left(h_{temp},h_{spat}\right)$$

The fused representation *z* is then mapped by a classification head to the anomaly probability $\hat{y}$.

#### 3.2.2. Kinematic Feature Representation

The temporal branch processes the four kinematic features at time step *t* defined by Equation (9), consistent with prior trajectory-based anomaly detection work in which speed, acceleration, and heading are extracted as kinematic descriptors [13]:

(9) $$x_{t}=\left[v_{t},a_{t},\sin\left(\theta_{t}\right),\cos\left(\theta_{t}\right)\right]$$

The segment identifier $id_{t}$ in Equation (2) is not an LSTM input; it selects the road-segment embedding extracted as $h_{spat}$. Directional information can distinguish maneuvers such as U-turns and detours [25]. Representing the heading angle $\theta_{t}$ through its trigonometric components prevents the discontinuity at the 0°/360° boundary, analogous to the sine–cosine encoding used for periodic temporal features in [21]. Each driving window derived from trajectory T is represented as $X\in R^{\tau \times 4}$ with τ=50, which serves as the input to the temporal branch in Equation (6).

#### 3.2.3. LSTM-Based Sequence Encoding

A two-layer stacked LSTM encoder, with 128 hidden units per layer and dropout of 0.2 between layers, updates its hidden state according to Equation (10) [23,26]:

(10) $$h_{t}=\mathrm{LSTM}\left(x_{t},h_{t-1}\right)$$

The final hidden state of the second layer serves as the temporal embedding, $h_{temp}=h_{\tau }$. Table 3 summarizes the branch. The encoder benchmark compares the LSTM with four temporal alternatives while holding the remaining components fixed.

#### 3.2.4. Graph-Based Contextual Features

The spatial module uses a GAT to encode the road network structure [9,11]. Each node $v_{i}$ is associated with a 17-dimensional structural vector $s_{i}$ capturing road-type descriptors, topological metrics (betweenness and closeness centrality), and contextual constraints (traffic-light indicators, tunnel information, rarity scores).

#### 3.2.5. Graph Attention Mechanism

The GAT layers compute attention coefficients over adjacent road segments using Equation (11) and aggregate the neighboring representations according to Equation (12) [24]:

(11) $$\alpha_{ij}=\frac{\exp\left(\mathrm{LeakyReLU}\left({\vec{a}}^{T}\left[Ws_{i}∥Ws_{j}\right]\right)\right)}{\sum_{k\in N_{i}}\exp\left(\mathrm{LeakyReLU}\left({\vec{a}}^{T}\left[Ws_{i}∥Ws_{k}\right]\right)\right)}$$

(12) $$h_{i}^{\prime }=\sigma \left(\sum_{j\in N_{i}}\alpha_{ij}Ws_{j}\right)$$

Two consecutive GAT layers are used: the first maps the 17-dimensional node attributes to a 64-dimensional intermediate representation (32 hidden channels, 2 attention heads); the second produces the final 64-dimensional spatial embedding. The embedding of the road segment occupied by the vehicle is extracted as $h_{spat}$. Table 4 summarizes the branch.

#### 3.2.6. Fusion Module

The temporal and spatial embeddings are projected into a shared latent space with dimension *d* = 64 according to Equations (13) and (14), respectively:

(13) $$\begin{array}{cc} h_{temp}^{\prime } & =\mathrm{ReLU}(W_{t}h_{temp}+b_{t}) \end{array}$$

(14) $$\begin{array}{cc} h_{spat}^{\prime } & =\mathrm{ReLU}(W_{s}h_{spat}+b_{s}) \end{array}$$

Four fusion operators are evaluated. The sample-wise scalar modality gate is defined by Equation (15) and combines the projected embeddings according to Equation (16):

(15) $$g_{gate}=\sigma \;\left(w_{2}^{⊤}\tanh\;\left(W_{1}\left[h_{temp}^{\prime }∥h_{spat}^{\prime }\right]+b_{1}\right)+b_{2}\right)$$

(16) $$z=g_{gate}\cdot h_{temp}^{\prime }+\left(1-g_{gate}\right)\cdot h_{spat}^{\prime }$$

where $W_{1}\in R^{32\times 128}$, $b_{1},w_{2}\in R^{32}$, and $b_{2}\in R$. This two-layer gate has 4161 parameters, consistent with Table 5. The output $g_{gate}\in \left[0,1\right]$ has tensor shape [B,1]: exactly one scalar per sample, broadcast across all 64 dimensions. The three comparison operators are fixed concatenation ($z=[h_{temp}^{\prime }∥h_{spat}^{\prime }]$, no reweighting), static weighted summation ($z=s\;h_{temp}^{\prime }+\left(1-s\right)\;h_{spat}^{\prime }$ with a single global learnable scalar *s*), and multi-head cross-attention (four heads, temporal embedding as query, both embeddings as key/value).

For a constant gate $\bar{g}$, a linear layer following fusion satisfies $Wz=\bar{g}Wh_{temp}^{\prime }+\left(1-\bar{g}\right)Wh_{spat}^{\prime }$. A classifier operating on concatenated embeddings can reproduce this transformation with separate weight blocks. This relation motivates the comparison with concatenation; it does not determine whether adaptive weighting improves predictive performance. Similarly, a static coefficient near 0.5 characterizes the fitted weighted-sum operator and provides no direct evidence about the value of sample-dependent fusion.

The fused representation passes through a two-layer MLP (64→32→1, ReLU, dropout 0.2) to produce the anomaly probability. Table 5 summarizes the stage.

### 3.3. Dataset and Preprocessing

The empirical evaluation uses physics-informed synthetic trajectories generated over OpenStreetMap (OSM) road networks. The primary dataset represents Kocaeli, Türkiye; independently generated Kocaeli and Hamburg, Germany, datasets support the replication experiments.

#### 3.3.1. Vehicle Trajectory Dataset

The evaluation uses synthetic trajectories with anomaly labels recorded during generation. Obtaining comparably annotated real sensor data is difficult because of privacy constraints, low anomaly prevalence, and limited verified incident annotations. A physics-informed trajectory generator is therefore used. Vehicle motion is simulated over the OSM-derived Kocaeli road network using a Markov-chain behavioral model in which each vehicle transitions between four kinematic states (constant speed, acceleration, deceleration, stop) at one-second resolution. State-transition probabilities are conditioned on the local traffic regime (free-flow, transition, congested), defined through a log-normal traffic-intensity distribution and the speed–density relationship in Equation (17), adapted from Pipes’ car-following model [27]:

(17) $${\bar{v}}_{s}=\left(v_{\max}-0.08v_{\max}\right){\left(1-\frac{\kappa }{10}\right)}^{4},$$

where $v_{\max}$ is the segment’s speed limit and κ∈[1,9] is the sampled traffic-intensity level. Acceleration is constrained to [−3, 1.5] m/s^2^ under normal driving, following AASHTO geometric-design guidance [28]. Sensor-level noise is injected into the speed and bearing channels at every time step.

The generated dataset contains 293,168 records across 90 inter-district trips. Ground-truth labels are recorded at injection time according to the taxonomy. A data card describing the generation settings, subtype rules, imputation proportions, and known limitations accompanies the code and data.

#### 3.3.2. Additional Kocaeli and Hamburg Datasets

Replication datasets are generated independently for Kocaeli and Hamburg using the same simulator family, anomaly taxonomy, and kinematic definitions. The Hamburg dataset contains 110 trips and 1183 distinct physical roads. Dictionary-based and KNN imputation define separate generation conditions in each city; their trajectories and labels differ, so between-dataset score differences do not isolate an imputation effect. Models are trained and evaluated within each dataset using five grouped folds and two seeds. This design assesses replication across road topologies and remains conditional on synthetic generation.

The underlying road topology for both cities is sourced from OpenStreetMap, with attribution to OpenStreetMap contributors. Vehicle trajectories and anomaly annotations are generated for this study. Models are retrained within Hamburg; this experiment does not evaluate transfer of a Kocaeli-trained model to that city.

#### 3.3.3. OpenStreetMap Road Network Data

Each road segment is represented in this study by a 17-dimensional feature vector $s_{i}$ comprising structural indicators (lane count, road category), infrastructure elements (traffic lights, tunnels), and topological measures (betweenness and closeness centrality).

Several structural attributes required imputation because of incomplete OSM tagging. Missing lane counts and speed limits were assigned road-type-specific defaults in Table 6; imputed speed-limit values were additionally smoothed according to Equation (18):

(18) $$\begin{array}{cc} {\hat{v}}_{i} & =\left\{\begin{array}{cc} {\mathrm{Round}}_{5}\;\left(\frac{v_{i-1}+v_{i}+v_{i+1}}{3}\right), & C_{i},\\ v_{i}, & \mathrm{otherwise}, \end{array}\right.\\ C_{i} & \equiv \left(S_{i}\neq \mathrm{OSM}\right)\wedge \left(\right|v_{i-1}-v_{i}\left|>30\right), \end{array}$$

The rule in Equation (18) removes physically implausible discontinuities in imputed limits. Traffic-light presence was inferred using a hybrid rule combining OSM traffic_signals tags with a node-degree heuristic.

The values in Table 6 are author-defined imputation assumptions, not observed OSM attributes or statutory limits: 53.8% of lane counts originate from OSM and 46.2% are imputed. Speed limits comprise 35.3% OSM values, 49.9% defaults, and 14.8% smoothed values. These imputation proportions are relevant to the spatial branch because $v_{\max}$ is included among its node features.

The primary Kocaeli network covers 10 central districts, with 90 inter-district routes forming the trip set.

#### 3.3.4. Graph Construction and Node Multiplicity

Both graph constructions are evaluated as prespecified experimental factors; held-out labels are not used to select between them. The trip-instance graph represents each trip-specific traversal of a road segment as a separate node, yielding 4678 nodes and 53,660 directed edges. These nodes correspond to 628 distinct physical roads; each physical road is represented by approximately 7.4 nodes on average. Features are identical within 538 of the 546 groups containing duplicate traversals. Merging nodes by physical-road identifier yields 628 nodes and 699 directed edges. The respective mean out-degrees, 11.47 and 1.11, describe these constructed graphs rather than the connectivity of the entire urban road network.

A directed edge connects segment *A* to segment *B* when the terminal OSM node of *A* matches the initial OSM node of *B*. This rule also creates cross-trip links between compatible segment copies. Edges do not depend on anomaly labels, trajectory co-occurrence, or learned similarities. Per-node label-purity ceilings computed from training labels are 94.58% and 92.84% for the trip-instance and merged graphs, respectively. These majority-label accuracy summaries describe label ambiguity and class imbalance. They are descriptive properties, not criteria for graph selection or bounds on achievable F1.

#### 3.3.5. Topology and Feature Access

The standard setting is transductive: the graph includes segments traversed by training, validation, and test trips, and GAT message passing operates over the complete graph during training. Node features are static road attributes and topological descriptors, not statistics computed from test anomaly labels. Nevertheless, graph membership reflects the observed route set, so these results do not represent training on an entirely unseen network. Scalers and class weights are estimated from training data only; checkpoint and threshold selection use the corresponding validation partition. Complementary controls restrict access to held-out segments, and independently generated datasets assess replication on a second road network.

#### 3.3.6. Empirical Transition Behavior

The generator’s transition probabilities and the procedure used to calibrate them are unavailable. To characterize the observed behavior, non-anomalous records are classified as stopped (v<1 km/h), accelerating (a>0.10 m/s^2^), decelerating (a<−0.10 m/s^2^), or approximately constant-speed observations. Transitions are counted between consecutive seconds within each trip. Mean speed decreases from 57.8 km/h under free flow to 25.0 km/h under congestion; the stopped-state share increases from 0.0001 to 0.0565. Acceleration and deceleration dominate the generated trajectories. Appendix B Table A2 reports the empirical transition matrices. These frequencies characterize the released trajectories but do not reconstruct the missing generator parameters.

#### 3.3.7. Data Cleaning and Feature Engineering

Of 293,168 generated records, 60 fall outside the speed range [0, 200] km/h and 8186 fall outside the road-gradient range [−50%, 50%]. One record falls outside both ranges, resulting in 8245 removed records (2.81%) and 284,923 retained records under the rule in Equation (19):

(19) $$\mathrm{retain}\;\mathrm{row}\;i\Leftrightarrow 0\leq v_{i}\leq 200\;\wedge \;-50\leq r_{i}\leq 50.$$

The heading angle is transformed into sine and cosine components to avoid the 0°/360° discontinuity.

#### 3.3.8. Anomaly Taxonomy and Label Generation

The dataset contains nine anomaly subtypes (Table 7). Anomaly prevalence is 6.86% before cleaning, 7.01% after cleaning, and 7.07% after windowing. These proportions refer to different stages of the pipeline.

The cleaning rule removes 55 of the 66 generated GPS speed artifacts, substantially reducing coverage of that subtype. Moreover, generation conditions are not sufficient to recover all labels: of 1400 cleaned records satisfying the speed-violation condition, 1353 carry that subtype, nine carry a GPS-artifact label, and 38 have no anomaly label. Subtype priority and additional injection conditions therefore affect the final annotations.

#### 3.3.9. Label–Input Dependence

Several subtype definitions use the kinematic channels supplied to the temporal branch, making the classification task partly dependent on its generating rules. Speed-limit violation additionally depends on $v_{\max}$, available through the node features. The label rule and model input use the same segment speed limit: the rule recovers all 13 speed-violation windows in the fixed test set, five of which lie on segments with imputed limits. High F1 on these data therefore reflects agreement with the synthetic labeling rules, rather than detection accuracy against independently verified traffic incidents. The rule-based reference and subtype analyses quantify this limitation.

#### 3.3.10. Feature Normalization, Sequences, and Partitioning

Acceleration is derived from one-second velocity differences, $a_{t}=\left(v_{t}-v_{t-1}\right)/3.6$ in m/s^2^, with velocity recorded in km/h. The two channels are therefore deterministically related. Velocity is normalized with Min–Max scaling and acceleration with Z-score standardization. Scalers are fitted on the training partition only and applied to validation and test data, avoiding the distributional leakage that arises when scaling precedes partitioning.

Trajectories are transformed into fixed-length sequences using a sliding window of *τ* = 50 steps with a stride of 10, yielding 28,090 sequences of which 7.07% are anomalous. Each window is assigned the anomaly label and road segment of its final time step.

The dataset is partitioned by unique trip identifiers rather than by individual samples, so that overlapping windows from the same route cannot appear in more than one partition. Two evaluation regimes are used:

- A fixed grouped partition (70/15/15 by trip) used for the encoder and fusion comparisons, summarized in Table 8.
- Five-fold trip-grouped cross-validation, evaluated with five seeds in the primary analysis and across repeated grouped partitions in the stability analysis. Every one of the 28,090 sequences receives an out-of-fold test prediction.

The lower test prevalence (3.27% versus 7.90% in training) reflects the anomaly composition of the trips assigned to each partition. Windows were not redistributed across partitions because doing so would violate trip-level grouping. This imbalance is one motivation for the cross-validated regime, where fold-wise test prevalence ranges from 3.40% to 10.49%.

### 3.4. Experimental Protocol

#### 3.4.1. Controlled Comparison Design

A run denotes a model fit for a specified configuration, partition, fold, and random seed. The main comparisons use Adam with learning rate 10^−3^, batch size 64, a maximum of 50 epochs, early-stopping patience 15, gradient clipping at max-norm 1.0, and ReduceLROnPlateau with factor 0.5 and patience 3. Encoder comparisons fix static weighted-sum fusion; fusion comparisons fix the LSTM and GAT branches. Operator-specific dimensions and initialization are documented in Appendix B Table A3.

#### 3.4.2. Loss and Checkpoint Selection

The implemented loss is the weighted binary cross-entropy defined by Equation (20), with the positive-class weight computed from training labels only:

(20) $$L=-\frac{1}{M}\sum_{i=1}^{M}\left[w_{pos}y_{i}\log\left({\hat{y}}_{i}\right)+\left(1-y_{i}\right)\log\left(1-{\hat{y}}_{i}\right)\right],$$

where $w_{pos}=N_{neg}^{train}/N_{pos}^{train}\approx 11.65$ on the fixed partition. Validation F1 is used for both checkpoint selection and early stopping in every model. Total training duration is reported separately from the epoch of the selected checkpoint. Termination before the epoch limit does not by itself establish optimal convergence.

#### 3.4.3. Run-Specific Decision Thresholds

Each model selects its decision threshold by maximizing F1 over a candidate grid on its own validation partition and applies that threshold unchanged to its test predictions. Under cross-validation, window *i* in fold *f* and seed *s* is classified according to Equation (21):

(21) $${\hat{c}}_{i,f,s}=I\;\left({\hat{p}}_{i,f,s}\geq t_{f,s}^{val}\right).$$

Thresholds are not averaged across folds or seeds. This prevents one fold’s test labels from influencing its decisions through thresholds selected in other folds. Each model retains its own operating point in paired comparisons. The available experiment documentation does not specify the candidate grid, which limits exact reproduction of the selected thresholds.

#### 3.4.4. Training Seeds and Repeated Partitions

The fixed partition and primary five-fold cross-validation use seeds 42–46, giving five and 25 runs per configuration, respectively. Seed 45, associated with a fixed-partition gate failure, is included. A complementary repeated-partition design crosses five grouped partitions, five folds, two shared seeds, five configurations, and two graphs: 500 graph–configuration runs, or 50 per cell. The graph-independent temporal control is shared across graph conditions. Additional independently generated datasets use five folds and two seeds. The number of seeds is specified for each design, and results from different designs are reported separately.

#### 3.4.5. Tuning, Initialization, and Procedural Consistency

The primary benchmark holds operator settings constant rather than exhaustively tuning each architecture. A follow-up applies the same learning-rate search to each tested configuration, using $\left\{5\times 10^{-4},10^{-3},2\times 10^{-3}\right\}$ with selection based on validation F1 and confirmation over five seeds. PyTorch and PyTorch Geometric default parameter initializations are used under the stated seeds. Each result is associated with a protocol hash covering the training, windowing, partitioning, backend, and library settings. Configurations reported in multiple analyses reuse the same cached runs.

#### 3.4.6. Statistical Units and Uncertainty

Length-50 windows with stride 10 share 80% of their time steps. Windows within a trip also share route and simulated motion conditions. The fixed test set contains 4799 windows drawn from 14 trip clusters, limiting the precision of trip-level inference. Cross-validation supplies out-of-fold predictions for all 90 trips, while dependence induced by shared training sets requires additional treatment.

#### 3.4.7. Prediction-Level Estimand

Pooled F1 is computed after applying the corresponding fold–seed threshold to each prediction. Pooled analyses are performed separately for each seed and retain the trip identifiers. Predictions for the same window across different seeds are dependent observations. Cluster-bootstrap intervals use 5000 trip resamples, retaining every window of each sampled trip. Two-sided permutation tests swap paired model predictions jointly within each trip over 20,000 permutations. These intervals and tests are conditional on the fitted models and do not capture the full variability across training seeds and partitions. Mean run-level F1 is the arithmetic average of run scores and differs from F1 computed after pooling contingency counts.

#### 3.4.8. Run-Level Estimand

F1 differences are paired between models evaluated on the same partition, fold, and seed. To account approximately for overlapping training sets, the corrected resampled variance is defined by Equation (22):

(22) $$\hat{\mathrm{Var}}\left(\bar{d}\right)=\left(\frac{1}{n}+\frac{n_{test}}{n_{train}}\right)S_{d}^{2},$$

where *n* is the number of paired runs and $S_{d}^{2}$ is the variance of their F1 differences. For the reported cross-validation design, $n_{test}=18$ and $n_{train}=59$ trips, giving a ratio of approximately 0.305. This correction widens uncertainty intervals but remains an approximation to dependence across folds, repetitions, and seeds.

Holm correction is applied within each declared comparison family. Architectural conclusions are based primarily on analyses that incorporate training variability. Conditional tests on pooled predictions are reported separately.

#### 3.4.9. Equivalence and Effect Sizes

A nonsignificant difference does not establish equivalence. The repeated-partition analysis also uses two one-sided tests with equivalence bounds of ±0.02 F1 and the corrected variance. This bound is evaluated separately from superiority testing and reflects the study’s measurement-resolution criterion, not an externally established practical margin. Paired per-trip F1 differences and standardized effect sizes are summarized for trips containing positive windows. Trips without positive windows contribute their false-positive counts to pooled inference.

#### 3.4.10. Evaluation Metrics and Implementation

Accuracy, precision, recall, F1, ROC-AUC, average precision, Brier score, and Expected Calibration Error (ECE) are reported where available. Experiments use PyTorch 2.7.1, CUDA 11.8, and PyTorch Geometric 2.7.0 on an NVIDIA RTX 4060 Laptop GPU with 8 GB memory.

## 4. Results and Discussion

The results proceed from fixed-partition comparisons to grouped evaluation and sensitivity analyses. Fixed-partition tables describe performance across training seeds on one held-out split; the cross-validation tables report variation across fold–seed runs, while pooled metrics summarize out-of-fold predictions for a specified seed. These summaries answer different questions and are interpreted separately. Across the corrected trip-level and run-level analyses, no comparison establishes a general architectural advantage. The temporal-only and classical baselines remain competitive with the hybrid, while sample-wise gating and cross-attention show occasional collapsed runs. The remaining analyses examine subtype performance, graph and feature choices, sensitivity to evaluation conditions, replication on additional synthetic networks, and computational cost.

### 4.1. Reference Points: Rule-Based and Classical Baselines

**Figure 2:**
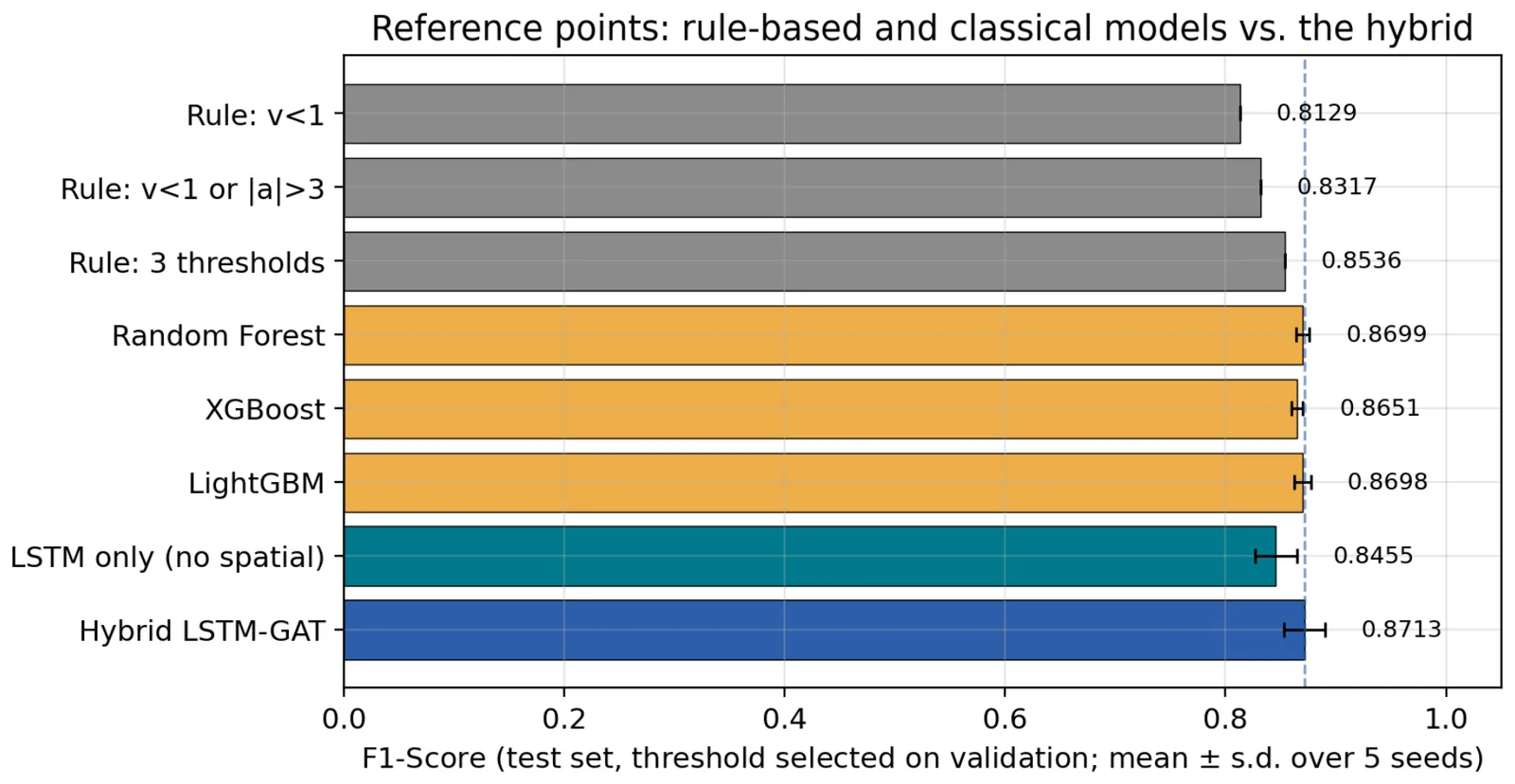
Fixed-partition baseline performance.

The fixed-partition references quantify how much of the synthetic task is recoverable from known generation rules or aggregated kinematic features. All learned models use training-only normalization and their own validation-selected thresholds. Table 9 and Figure 2 compare these references on 4799 test windows, including 157 positives; the hybrid uses static weighted-sum fusion.

The three-threshold rule reaches F1 0.8536, compared with 0.8713 ± 0.0188 for the weighted-sum hybrid. Random Forest differs from that hybrid by only 0.0014 in mean F1 and has higher ROC-AUC (0.9964 versus 0.9638). These results are consistent with substantial label–input dependence: simple rules and aggregates already capture much of the task. The trip-level comparisons do not establish a performance advantage for the hybrid. Classical baselines are also evaluated under grouped cross-validation.

### 4.2. Encoder Benchmark

**Figure 3:**
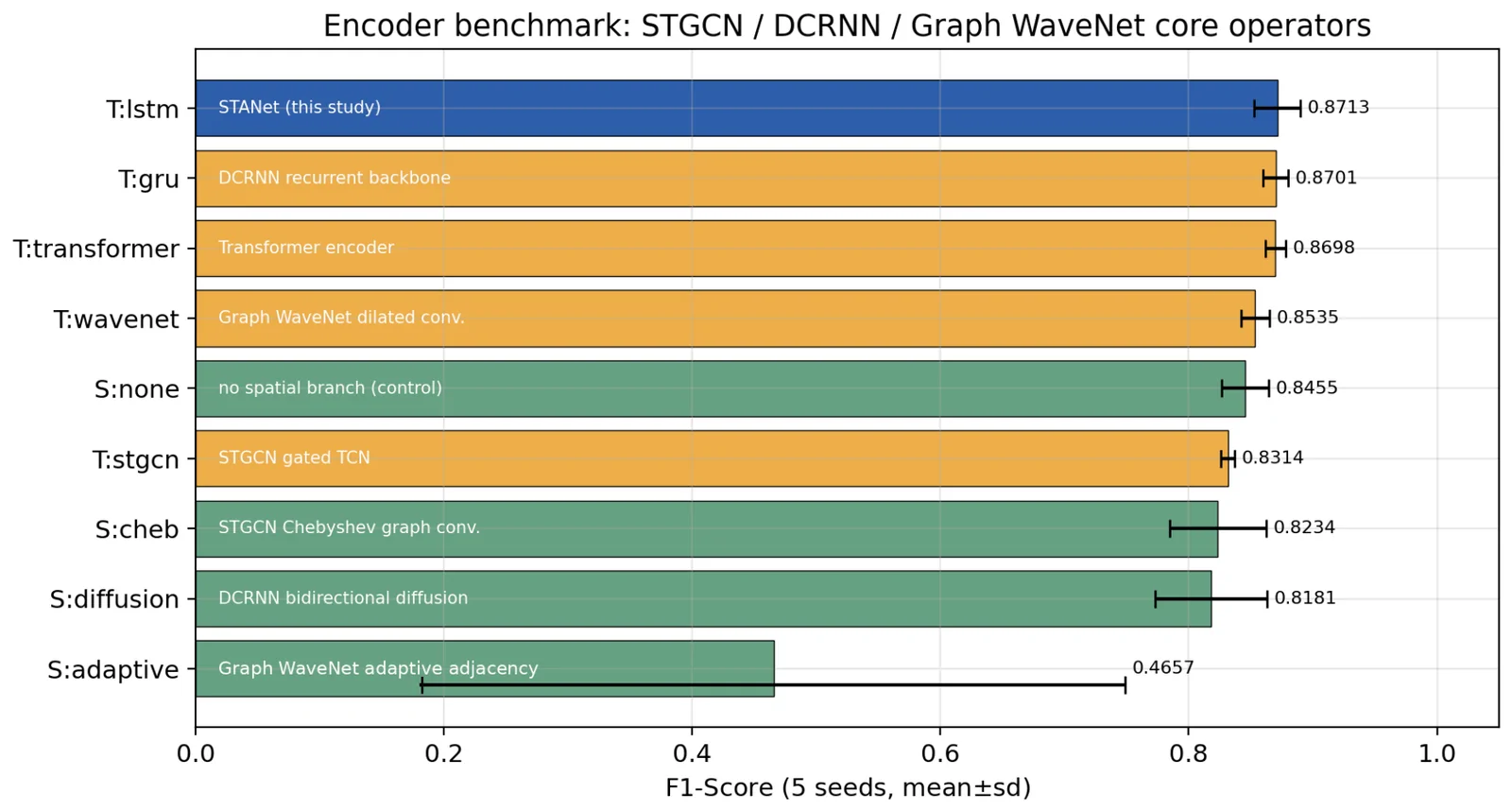
Fixed-partition encoder F1.

STGCN [29], DCRNN [30], and Graph WaveNet [31] were designed for graph-wide traffic forecasting. The present task instead classifies a single vehicle window associated with a road segment. We therefore adapt their core operators rather than benchmark their complete published architectures. Temporal alternatives include a GRU, gated temporal convolution, dilated causal convolution, and a Transformer. Spatial alternatives include Chebyshev convolution, bidirectional diffusion convolution, and adaptive adjacency; a temporal-only model serves as the control. Static weighted-sum fusion and the classification protocol remain fixed. Table 10 and Figure 3 summarize F1 as the mean ± standard deviation over five training seeds under the common learning rate. The table reports unadjusted window-level *p*-values; inferential conclusions use the trip-level analysis.

The LSTM–GAT, GRU–GAT, and Transformer–GAT configurations span only 0.0015 in mean F1, compared with a seed standard deviation of 0.0188 for LSTM–GAT. None of the eight fixed-partition encoder comparisons meets the trip-level Holm-corrected significance criterion. The reported ranking therefore establishes neither temporal-encoder nor spatial-operator superiority. The adaptive-adjacency configuration exhibits high variability under the common learning rate (0.4657 ± 0.2833).

The validation-based learning-rate search retains 10^−3^ for seven of eight tested encoder configurations. Adaptive adjacency selects 5 × 10^−4^ and reaches 0.8113 ± 0.0719 F1. This substantial change indicates sensitivity to optimization settings; its untuned result should not be attributed solely to the operator. Appendix B Table A4 reports selected rates and best-checkpoint epochs. The prediction-level tests of the common-rate benchmark apply only to that benchmark and are not transferred to the tuned results.

### 4.3. Fixed-Partition Fusion Ablation

With both encoders and the remaining protocol held fixed, Table 11 reports fusion F1 as the mean ± standard deviation over five seeds. Static weighted summation has the highest mean F1, while the sample-wise gate has the largest variability. One gated run at seed 45 reaches F1 0.0715, with mean gate values 0.026 overall and 0.0125 on anomalous windows. A comparable failure recurred under a different backend configuration. None of the 15 fixed-partition runs of the other operators meets the degeneracy criterion of F1 <0.30.

The fitted static weights are {0.519,0.522,0.518,0.498,0.497}. Their proximity to equal weighting characterizes the fitted static operator; it does not determine whether sample-dependent fusion is beneficial. Likewise, the difference in mean performance does not establish operator superiority after trip-level correction. Broader stability evidence is provided by the repeated-partition analysis below.

### 4.4. Gate Behavior and Training Diagnostics

**Figure 4:**
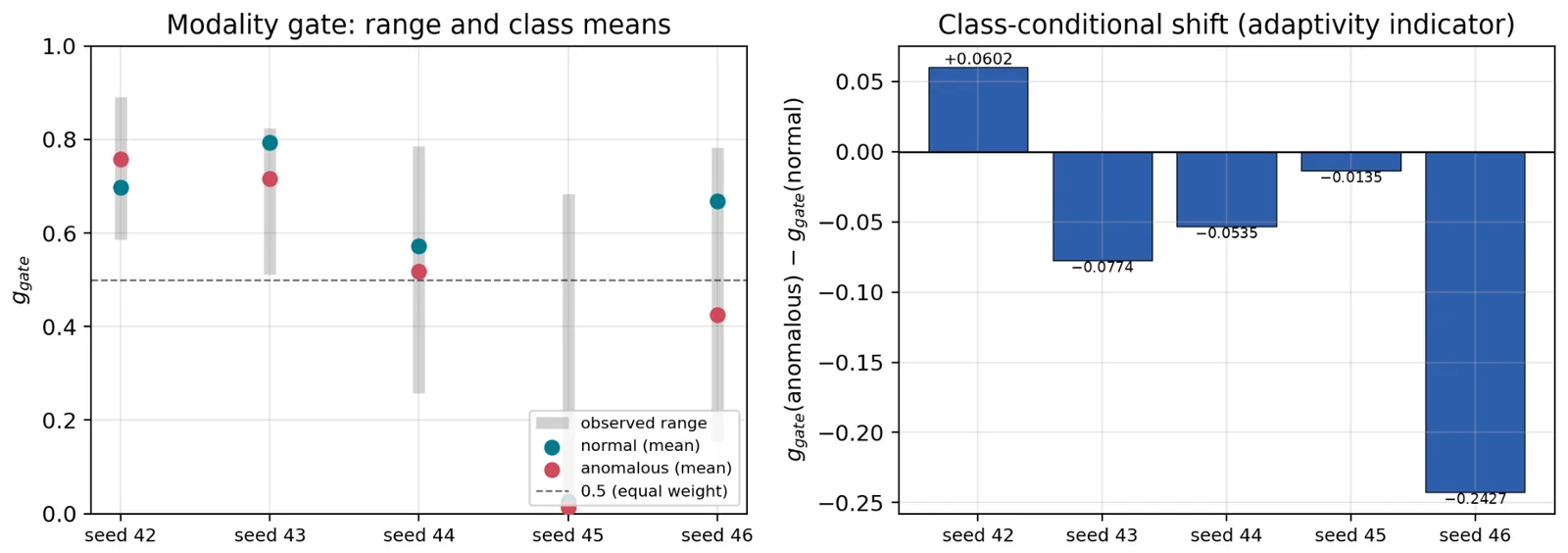
Gate distributions across training seeds.

Figure 4 shows the gate distributions across five fixed-partition seeds. The differences between mean gate values for anomalous and normal windows are +0.0602, −0.0774, −0.0535, −0.0135, and −0.2427. Both sign and magnitude vary, so the gate does not support a stable class-conditional interpretation.

Epoch-level diagnostics cover ten runs, five per graph. In nine, the validation-set mean gate crosses 0.5 between epochs 4 and 19, and final F1 ranges from 0.7557 to 0.8742. In the remaining run, the mean gate peaks at 0.3815, never assigns greater mean weight to the temporal branch, and training ends at epoch 18 with F1 0.0759. Early negative class-conditional shifts also occur in successful runs, so their sign alone does not explain failure.

The observed gate behavior is consistent with suppression of the temporal branch. Treating the gate as fixed, Equation (16) gives $\partial z/\partial h_{temp}^{\prime }=g_{gate}I$; this direct path weakens as the gate approaches zero. The complete derivative also includes the gate’s dependence on both embeddings. This interpretation remains a mechanistic hypothesis requiring quantitative embedding and gradient diagnostics.

### 4.5. Cross-Validated Fusion Evaluation

Five trip-grouped folds crossed with seeds 42–46 provide 25 runs per configuration and out-of-fold predictions for all 90 trips. Each prediction uses its corresponding validation-selected threshold. Table 12 reports run-level F1 as the mean ± standard deviation across the 25 runs. Pooled F1, Brier score, and ECE use seed-42 out-of-fold predictions. A run is classified as collapsed when F1 is below 0.30.

The fusion ranking changes with the number of training seeds: gating has the highest hybrid mean in the two-seed analysis (0.8908±0.0429), but falls below weighted summation and concatenation when five seeds are included. Gating and cross-attention each have one degenerate run in the expanded design. The full-run hybrid means span 0.0281 using the rounded values in Table 12; a supplementary summary excluding degenerate runs spans approximately 0.0075. The analysis excluding degenerate runs describes performance conditional on successful training; the full-run analysis remains the primary evaluation.

Conditional seed-42 trip-level tests compare gating with weighted summation (ΔF1=−0.0042, 95% CI [−0.0162,+0.0102], Holm p=1.000), cross-attention (+0.0011, [−0.0120,+0.0175], Holm p=1.000), and concatenation (+0.0321, [+0.0052,+0.0689], Holm p=0.099). None establishes a pairwise fusion advantage after correction. These pooled differences condition on one seed and must be distinguished from the 25-run means. Repeating the pooled analysis with the second seed reverses the sign of three of the four differences between a hybrid and the temporal-only control, illustrating their sensitivity to training variability.

#### Controlled Replication

Repeating five fold-specific runs under an identical seed and protocol produces exact agreement at reported precision for the temporal-only control. For LSTM–GAT, none of the five pairs agrees, with mean absolute F1 difference 0.0195 and maximum 0.0414. This implementation-specific replication result supports reporting multiple training runs in the present setting. It does not establish irreproducibility across all GAT implementations.

### 4.6. Repeated Grouped Partitions and Graph Stability

Five grouped partitions are constructed by permuting trip identifiers with partition-specific seeds and dividing them into five blocks; the reference partition reproduces the initial grouped split. Crossing partitions, folds, two shared training seeds, five configurations, and two graphs produces 50 runs per graph–configuration cell. Table 13 reports mean F1 ± standard deviation and counts of degenerate runs (F1 <0.30). The temporal-only control is graph-independent and shared across the two graph conditions.

With corrected resampled inference and Holm adjustment, none of the 20 pairwise comparisons, ten per graph, is significant. None satisfies the reported equivalence criterion of ±0.02 F1 either. Thus, the design establishes neither superiority nor equivalence. The mean F1 difference between weighted-sum fusion and the temporal-only control is +0.0049 on the trip-instance graph and approximately +0.0016 on the merged graph; both intervals include zero.

All eight degenerate graph–configuration runs occur in the sample-wise gate or cross-attention: four of 100 for each operator. Static weighted summation, which also learns a coefficient, has no degenerate runs. The observed failures are associated with sample-dependent weighting, whereas the model with a learned global coefficient has no degenerate runs in this experiment. The 4% observed rates have limited precision and do not establish general failure probabilities. Three gated failures occur on the merged graph, indicating that the absence of collapse in the five-seed fixed-partition comparison does not generalize to repeated partitions.

A descriptive variance decomposition attributes, on average, 8.6% of F1 variation to partition, 7.6% to fold, and 0.9% to seed. The remainder combines interactions and residual variability, which cannot be separated with one observation per cell. This decomposition supports evaluation across multiple partitions, although the variance components do not identify causal sources of training noise.

#### Subtype Stratification

Subtype metrics use all out-of-fold seed-42 predictions, comparing static weighted-sum LSTM–GAT with LSTM-only. For subtype *k*, positives are windows carrying label *k* and negatives are normal windows; windows of other anomalous subtypes are excluded. The shared negative set yields 89 hybrid false positives in every row. Consequently, subtype precision and F1 reflect both subtype detection and this common false-positive burden.

All nine subtypes are reported in Table 14. Inferential testing is restricted to subtypes with at least 30 positive windows and five positive trips, leaving four comparisons. Holm adjustment is applied to the permutation *p*-values for these four comparisons. In Table 14, P and R denote hybrid precision and recall. GPS speed artifacts survive cleaning at the record level but yield no positive labeled windows after windowing.

After run-specific thresholding, the abnormal-dwell difference is +0.0074 F1, with a trip-bootstrap interval approximately [−0.0080,+0.0265]. A significant gain of +0.0335 appears only when pooled thresholds are used and is not supported after run-specific thresholding. Speed-limit violation also has an interval spanning zero (approximately [−0.3044,+0.1152]). Signal freeze favors the temporal control (Δ=−0.0587; unadjusted p≈0.043), but does not survive correction. None of the eligible subtypes demonstrates a spatial-branch benefit in this conditional analysis, which does not establish absence of benefit. The abnormal-dwell estimates are conditional on the fitted training seed and do not establish a reproducible advantage across seeds. A multi-seed subtype analysis is not available, and the interval does not capture variability across fitted models.

### 4.7. Contextual Features, Message Passing, and Held-Out Segments

Three controls assess the separate contributions of node attributes and graph propagation. Each is trained from scratch: retaining self-loops only preserves node features while removing inter-node propagation; shuffling feature rows breaks their association with road segments; random edges of matched density preserve graph size while discarding physical connectivity. Table 15 reports five-fold, five-seed comparisons (25 runs per condition). Differences are defined as control minus weighted-sum reference; confidence intervals and Holm-adjusted *p*-values use paired run-level F1 with the overlap correction in Equation (22).

None of the comparisons establishes a predictive benefit from the original features or connectivity. The wide intervals also leave equivalence with an arbitrary graph unresolved. Selected single-seed pooled tests favor perturbed controls, but those conditional results do not replace the multi-seed inference.

In the held-out-segment control, nodes appearing only in test trips are isolated and their feature rows are set to zero during training, affecting 595–657 trip-instance nodes per fold. Nodes shared with training or validation trips remain visible; the full graph is restored at inference. Weighted-sum F1 changes from 0.8839±0.0415 to 0.8964±0.0362 (Δ=+0.0125, 95% CI [−0.0218,+0.0467]), and gated F1 from 0.8589±0.1458 to 0.8763±0.0478 (+0.0174, [−0.1595,+0.1944]). Both Holm-adjusted *p*-values are 0.918. This control evaluates restricted access to held-out segments within the same observed network. It does not provide fully inductive graph evaluation or cross-city transfer validation.

### 4.8. Comparison with Classical Baseline Models

Classical baselines use 38 window aggregates and the same grouped folds and validation-threshold policy as the neural models. The three-threshold rule is also evaluated within this regime. Table 16 summarizes run F1 as the mean ± standard deviation over five folds and five seeds (25 runs). Pooled F1 and paired trip-level tests against the gated hybrid use seed-42 predictions for all 90 trips. The rule has no trainable parameters, so its repeated seed entries do not represent distinct fitted models.

The pooled F1 difference between LightGBM and the gated hybrid is +0.0409 F1 (95% trip CI [+0.0278,+0.0552], Holm p=0.0002). The available paired run-level comparison covers ten fold–seed runs from the earlier two-seed subset: +0.0272, corrected interval [−0.0106,+0.0650], Holm p=1.000. Run-level inference is therefore restricted to this subset, whereas the descriptive summaries in Table 16 cover 25 runs; a paired analysis of the complete five-seed design remains unreported. The analyses address different uncertainties: the pooled test conditions on fitted predictions, whereas the run-level test includes training variation and overlapping training sets. Accordingly, the classical baselines are competitive, but the available run-level analysis does not establish a performance advantage for either the hybrid architecture or the classical comparator.

### 4.9. Sensitivity to Windowing, Prevalence, Noise, and Imputation

Eight combinations of window length (25, 50, or 100 steps) and stride (10, 25, or 50) cover overlap from 0% to 90%. Each condition has its own protocol hash. No comparison between the weighted-sum hybrid and the temporal-only control is significant under corrected paired inference (Appendix C Table A5). The sensitivity experiment’s length-50, stride-10 reference has F1 0.8865±0.0360 for the hybrid and 0.8871±0.0468 for the temporal control. These runs belong to a separate sensitivity experiment and differ from the cached 25-run reference in Table 12. Each windowing condition uses five grouped folds and one training seed, giving five runs per model; the reported standard deviation therefore summarizes variation across folds rather than across seeds. The seed identifier is not specified in the available documentation.

Prevalence sensitivity is evaluated analytically using fold-specific true-positive and false-positive rates from the two-seed experiment. Models are not retrained, and anomaly intensities are not independently regenerated. For prevalence π, true-positive rate *r*, and false-positive rate *q*, F1 is defined by Equation (23):

(23) $$F1\left(\pi \right)=\frac{2\pi r}{\pi (1+r)+(1-\pi )q}.$$

Under this fixed-rate assumption, weighted-sum F1 increases from 0.730 at 1% prevalence to 0.907 at 25% (Appendix C Table A6). These results quantify F1 sensitivity to class prevalence under fixed detection rates; they do not assess robustness to changes in the traffic-generating process.

White-noise and AR(1) speed perturbations are applied at inference to the trained models, with decision thresholds held fixed. Perturbed speed ${\tilde{v}}_{t}=v_{t}+\varepsilon_{t}$ is propagated to acceleration by recomputing ${\tilde{a}}_{t}=\left({\tilde{v}}_{t}-{\tilde{v}}_{t-1}\right)/3.6$ at the one-second sampling interval; independent acceleration noise is not added. Weighted-sum F1 decreases from 0.904 without added noise to 0.034 under white noise with σ=10 km/h, or 0.448 under AR(1) noise with ρ=0.9 (Appendix C Table A7). Temporal correlation changes the perturbations induced by differencing speed. The number and identifiers of the fitted models used in this sensitivity experiment are not documented, so these results remain descriptive and do not quantify variability across training seeds.

Imputation controls are retrained under the primary five-fold, five-seed design: adding missing-value indicators gives F1 0.8755±0.0634, replacing defaults with road-type medians gives 0.8821±0.0484, and removing speed-limit and lane-count features gives 0.8774±0.0436, versus 0.8839±0.0415 for the reference. Corrected intervals include zero in all cases (Appendix C Table A8). These feature interventions hold the original trajectories and labels fixed; they do not test how alternative speed-limit assumptions would change anomaly generation.

### 4.10. Replication on Independently Generated Road-Network Data

Additional synthetic datasets are generated for Kocaeli and Hamburg using dictionary-based or KNN imputation of road attributes. Hamburg contains 110 trips and 1183 distinct physical roads. Models are trained and evaluated within each dataset using five folds and two seeds; this is replication on another network rather than transfer of a Kocaeli-trained model to Hamburg. Table 17 summarizes ten pairwise comparisons among five configurations per dataset, with equivalence bounds of ±0.02 F1.

A separate audit examines dependence between labels and features in the additional datasets. After their prescribed filtering, every tunnel record carries a signal-freeze label, making tunnel presence directly predictive of that subtype. The tunnel-associated share of anomalies is 60.4% in regenerated Kocaeli and 5.1% in Hamburg. Paired feature ablations remove the tunnel indicator while retaining trajectories and labels. On the Hamburg network, the gated hybrid has F1 0.8664±0.0383 without the indicator and 0.6435±0.2931 with it; cross-attention changes from 0.8829±0.0300 to 0.8246±0.1755. These descriptive results show that a label-linked attribute can alter training stability, but they do not establish the causal mechanism of the failures. Both settings are trained within Hamburg; their difference assesses an attribute intervention on that network rather than cross-city transfer.

None of the 40 within-dataset pairwise comparisons is significant after Holm correction, while the reported equivalence outcomes vary by dataset. Six runs in the noisiest dataset are degenerate, all involving gating or cross-attention. These results extend the evaluation to different road topologies, while remaining conditional on one simulator and anomaly taxonomy. Nominal prevalence targets of 1%, 5%, and 15% yield realized rates averaging 16.4%, 20.8%, and 24.7%; intended noise levels are also insufficiently separated. Thus, the generated variants do not provide a controlled independent-dataset test of those factors. The imputation strategies are applied to separately generated trajectories and labels; their comparison therefore does not constitute a paired feature intervention.

### 4.11. Per-Trip Performance and Effect Sizes

Of 90 trips, 39 contain positive windows. Table 18 summarizes paired F1 differences for those trips, while the 51 negative-only trips remain in pooled inference through false positives. Per-trip F1 summaries are restricted to trips containing positives because recall is undefined for trips without positives. F1 for such trips depends on the handling of zero denominators. The standardized effect size $d_{z}$ is the mean paired difference divided by its standard deviation; counts favoring either model exclude ties. These effect sizes describe single-seed out-of-fold predictions and are not estimates of variability across fitted models.

The distributions contain effects in both directions. For weighted sum versus LSTM, 18 trips favor the hybrid, 14 favor the control, and seven are tied; one difference of −0.4444 substantially influences the mean. Mean per-trip and pooled F1 differences apply different weights to the observations and represent distinct summaries.

### 4.12. Calibration

**Figure 5:**
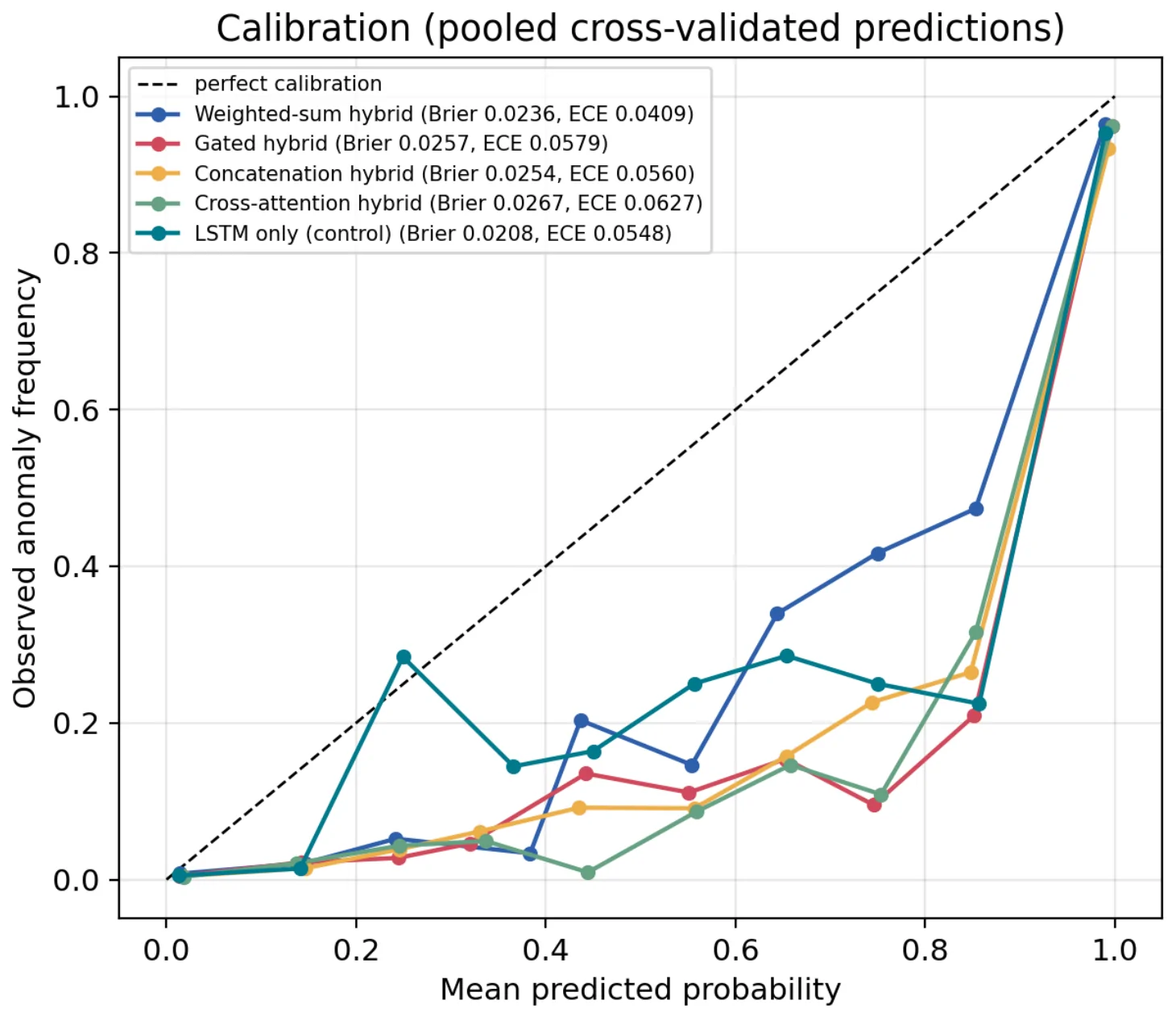
Out-of-fold probability calibration.

The reliability diagram in Figure 5 uses seed-42 pooled out-of-fold probabilities. These probabilities give the weighted-sum hybrid the lowest hybrid ECE (0.0409), while LSTM-only has the lowest Brier score (0.0208). Because these metrics use predicted probabilities directly, correcting the classification thresholds does not change them. The estimates are conditional on one training seed and do not establish a stable calibration advantage. Using the predictions as risk scores requires further calibration assessment and validation in the target population.

### 4.13. Fixed-Partition Graph Construction Comparison

Table 19 presents the fixed-partition graph comparison as a supplementary analysis, with F1 reported as the mean ± standard deviation over five seeds and the fusion operator specified for each hybrid configuration. Standalone GAT ROC-AUC is higher on the merged graph, but F1 remains low on both. Hybrid performance varies with fusion operator and graph representation.

No gated failure occurs among the five merged-graph fixed-partition runs. The repeated-partition experiment nevertheless records three such failures on that graph (Table 13). Thus, the fixed-partition result neither establishes that merging prevents collapse nor identifies a general graph-construction advantage.

### 4.14. Fixed-Partition Inference at the Trip Level

The fixed test set contains 14 trips, with 75–760 windows per trip. Table 20 distinguishes window-level McNemar tests from trip-level F1 permutation tests based on joint swaps of paired predictions within trips. Because these tests differ in both statistical unit and test statistic, their contrast does not isolate the effect of changing the unit alone. F1 differences and uncertainty intervals are computed for the median seed, and trip-level *p*-values receive Holm correction within each comparison family. The rule comparator is a one-threshold dwell-only classifier, in contrast to the three-threshold reference classifier. The widening column gives the ratio of trip-level to window-level interval width.

None of the 16 trip-level comparisons meets the Holm-adjusted 0.05 criterion. The median-seed weighted-sum F1 is 0.8851, with trip-bootstrap interval [0.7231,0.9470]. Interval-width ratios range from 0.85 to 2.79; clustering does not widen every observed interval. For adaptive adjacency, the unadjusted *p*-value is 0.007 and the adjusted value is 0.056; the comparison therefore does not meet the prespecified significance criterion.

A fixed-partition trip-level comparison with the three-threshold rule remains unreported; the dwell-only row does not provide this comparison. Because the statistical unit, test statistic, and multiplicity adjustment differ, these results compare evaluation protocols and do not isolate the effect of within-trip dependence.

### 4.15. Computational Efficiency and Edge Feasibility

**Figure 6:**
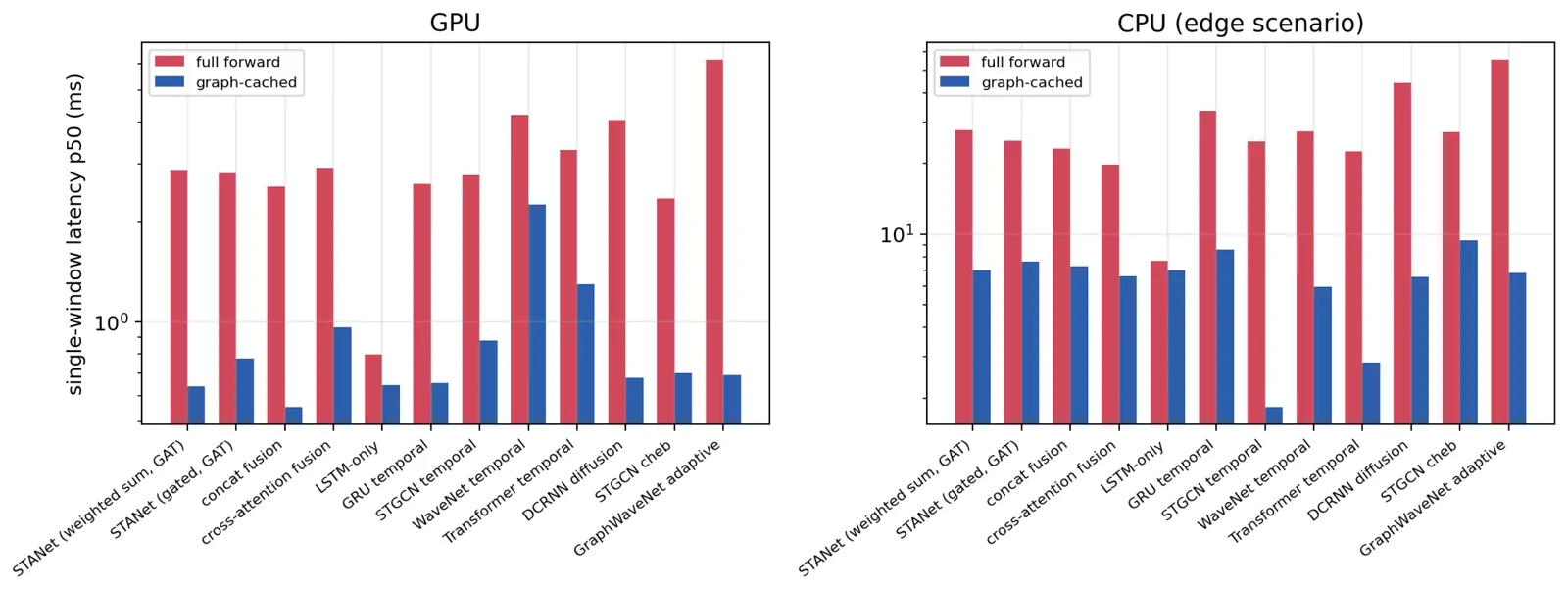
GPU and CPU inference latency.

Table 21 reports parameter counts and median single-window inference latency measured with batch size 1, after 50 warm-up iterations, over 500 repetitions on GPU and 60 on CPU. Measurements use the trip-instance graph (4678 nodes). Figure 6 compares GPU and CPU latency with and without graph-embedding caching on a logarithmic scale.

Table 22 reports fusion training cost over five fixed-partition seeds on the RTX 4060 Laptop GPU. Epoch counts and elapsed times are means; parameter counts distinguish the fusion operator from the complete model.

The weighted-sum model contains 220,802 parameters (≈0.9 MB in fp32), of which the temporal branch accounts for 200,704 (90.9%), the projection layers 12,416, the classifier 2113, the spatial branch only 5568 (2.5%), and the fusion operator a single scalar. Peak GPU memory is 66 MB.

Despite its small share of the parameters, the spatial branch accounts for most of the inference cost when embeddings for all 4678 nodes are recomputed at every forward pass. Because the road network is static, node embeddings can be computed once and cached, reducing GPU latency from 2.87 to 0.64 ms (4.5×) and CPU latency from 27.82 to 7.03 ms (4.0×). For static weighted-sum LSTM–GAT, the median cached latency equals the corresponding LSTM-only value at the reported precision on the measured GPU and CPU (0.64 ms and 7.03 ms, respectively). This agreement applies to the tested configuration, hardware, and measurement protocol. The different cached latencies of the other fusion operators in Table 21 preclude a general claim of zero inference overhead.

On the tested CPU, weighted-sum inference has a 95th-percentile latency of 39.92 ms without caching and 8.77 ms with caching. These measurements support the computational feasibility of the tested implementation; throughput on an edge device requires separate hardware-specific evaluation.

### 4.16. Scope of the Contribution

The contribution is an empirical assessment of how partitions, training seeds, fold-specific thresholds, graph representations, and uncertainty estimation affect architectural conclusions. Increasing the number of training seeds reveals failures that were absent from the smaller design. Replacing pooled thresholds with run-specific thresholds removes the apparent abnormal-dwell improvement, while accounting for training variability limits the generalization of single-seed pooled differences. These results concern evaluation practice rather than the introduction of a new architecture or inferential method.

The additional hybrid capacity provides no established general advantage over temporal-only or classical references under the available run-level analyses. This conclusion is specific to the synthetic generators and evaluated implementations. Labels derived from generation rules may favor kinematic representations and tabular aggregates. Matching the comparison protocol does not remove this limitation from conclusions about relative architectural performance. The empirical findings therefore motivate stronger evaluation protocols rather than a universal ordering of fusion methods.

### 4.17. Threats to Validity

#### 4.17.1. External Validity and Label Dependence

All trajectories are simulated, including the additional network datasets. The use of one generator family and a nine-subtype taxonomy limits the range of conditions represented. Several labels depend directly on input channels or road attributes, and filtering removes many GPS speed artifacts. In the regenerated data, the tunnel indicator is label-linked after preprocessing. These properties affect both absolute performance and relative architectural findings; real sensor data and independently verified incidents are required for external validation.

#### 4.17.2. Generation and Sensitivity Specification

Empirical transition matrices characterize the realized behavior of the released trajectories. The nominal prevalence and noise settings in the generated variants do not yield adequately controlled differences between conditions. Analytical prevalence reweighting, perturbations applied at inference, and imputation controls with fixed labels assess narrower aspects of sensitivity than regeneration under changed traffic conditions. Windowing sensitivity uses five folds and one training seed. In the noise experiment, acceleration is recomputed from perturbed speed. The supplementary results characterize sensitivity under the reported settings. Incomplete documentation of transition calibration, the threshold-search grid, seed records, and fitted-model counts limits exact reproduction of these analyses and selected operating points.

#### 4.17.3. Statistical Precision and Estimands

The fixed test set has 14 trip clusters, and the primary dataset has 90 trips, only 39 of which contain positive windows. Pooled tests are conditional on fitted models, whereas corrected run-level tests provide an approximate treatment of additional training variability. Overlapping training sets and reused trips remain sources of dependence. Neither nonsignificance nor small point estimates establish equivalence. The reported equivalence bound is a study-specific resolution criterion, and its suitability for practical traffic decisions requires independent justification.

#### 4.17.4. Adaptation, Tuning, and Transductive Access

STGCN, DCRNN, and Graph WaveNet are represented by adapted operators rather than their complete forecasting systems. The learning-rate search is limited and does not establish that every baseline is optimally tuned. The standard graph is transductive, and the held-out-segment control retains shared-road information and restores the full graph at inference. Training models independently on Hamburg assesses replication across road topologies. It does not evaluate transfer of a trained model to an unseen city.

## 5. Conclusions and Future Work

This study evaluates hybrid LSTM–GAT components using matched training protocols, grouped partitions, shared seeds, and separately reported prediction-level and run-level inference. Fixed-partition comparisons do not establish superiority after trip-level Holm correction. Five-seed cross-validation reveals occasional degenerate runs in gating and cross-attention, while repeated grouped partitions show that such failures can occur on either graph construction. The observed rates characterize the evaluated runs rather than general operator reliability.

Applying the corresponding fold–seed threshold to each prediction removes the apparent improvement in abnormal-dwell detection observed under pooled thresholds. Contextual-feature and edge controls likewise do not establish a spatial-branch contribution under corrected run-level inference. Classical references remain competitive, and the significant single-seed pooled comparisons must be interpreted alongside the nonsignificant run-level analysis based on fewer training runs. Replication across additional synthetic network datasets extends these findings beyond the original topology while retaining generator dependence.

### 5.1. Implications

Comparative studies with overlapping windows should report the clustering structure, distinguish mean run F1 from pooled F1, apply each validation threshold only to its corresponding test run, and quantify partition and training variation. Conditional prediction-level uncertainty is useful but cannot substitute for variability across fitted models. Simple rule and classical references are particularly informative when labels are generated from known input relationships. Computational measurements should state the graph size and caching policy alongside accuracy results.

### 5.2. Future Directions

Future work should prioritize independently annotated sensor trajectories and context-dependent anomaly generation, with controlled variation in traffic regimes, anomaly intensity, and measurement noise. Generator parameters and preprocessing rules should be released together, and preprocessing should retain the artifacts targeted by anomaly detection. Cross-city transfer requires training on one network and testing on another, rather than separate within-network training.

Gate-stabilization strategies, including bounded coefficients, entropy penalties, or auxiliary branch supervision, require controlled experiments with embedding and gradient diagnostics. Broader baseline tuning and larger samples of independent trips would also improve the precision of superiority tests and equivalence assessments based on practically justified margins.

Real-time deployment may further require reducing the volume of incoming GPS points before kinematic features are extracted. Candidate approaches include classical trajectory simplification, such as the Ramer–Douglas–Peucker algorithm [32], time-ratio methods [33], and spatiotemporal sampling [34], as well as ANN-based [35] and window-based [36] reduction. Because these methods discard trajectory points, they alter the speed, acceleration, and heading values within each driving window and may remove brief maneuvers, such as U-turns and sudden braking, that characterize short-duration anomalies. Their effects on detection sensitivity and false-positive rates should therefore be evaluated separately before they are integrated into the proposed framework.

## Figures and Tables

**Table 1:** Compact comparison of selected studies and the present evaluation.

Study	Task and Data	Evaluation Design	Uncertainty and Reported Finding
Huang et al. [4]	Multivariate time-series anomaly detection; SWaT, SMAP, MSL, and SMD	Predefined train/test split; five runs; no CV	Uncertainty not reported; about two percentage points higher F1 than the strongest baseline
Duan et al. [5]	Highway anomaly detection; METR-LA and PEMS-BAY	Random 80:20 split; 1000 simulation trials averaged; no CV	Significance described qualitatively; F1 of 0.967 versus 0.945 for static detection
Nguyen et al. [6]	Traffic-volume and speed forecasting; Dublin2021 and six further datasets	70/10/20 split; five runs for the proposed model only	SD reported for the proposed model only; paired CI not reported; up to 12.3% lower MAE
Liang et al. [7]	Network intrusion detection; BoT-IoT, TON_IoT, and UNSW-NB15	Partition not reported; CV for teacher only	*t*-test with p<0.05; test details not reported; 99.28% accuracy on UNSW-NB15
Cui et al. [8]	Trajectory prediction; NGSIM and HighD	7:1:2 split; test-scene vehicles excluded; no CV	Wilcoxon test; pairing unit not reported; RMSE reduced by 0.27–0.80 m on NGSIM
This study	Traffic anomaly detection; 90 synthetic trips	Trip-grouped split; five-fold CV; five repeated grouped partitions	Trip-bootstrap CI, trip-level permutation, and corrected resampled tests; neither superiority nor equivalence established

Notes: CV: cross-validation; SD: standard deviation; CI: confidence interval; MAE: mean absolute error; RMSE: root mean squared error.

**Table 2:** Mathematical notation.

Symbol	Description
G =(V,E)	Directed graph representing the road network
V,E	Set of nodes (road segments) and edges (connectivity)
N,A	Number of nodes and adjacency matrix encoding connectivity
$s_{i},S$	Structural feature vector of node *i* and the set of all structural features
$T=\{x_{1},\ldots ,x_{\tau }\}$	Vehicle trajectory sequence of length τ
$x_{t},X\in R^{\tau \times 4}$	Per-step kinematic vector and stacked temporal feature matrix
$v_{t},a_{t},\theta_{t},id_{t}$	Velocity, acceleration, heading angle, and road-segment ID at time *t*
$x_{sin},x_{cos}$	Trigonometric heading components used for continuous angular representation
$type_{i},length_{i}$	Road category and segment length;
$deg_{i},context_{i}$	node degree and contextual attributes
$h_{t},h_{temp},h_{spat}$	LSTM hidden state, temporal embedding, and spatial embedding
$\Theta_{temp},\Theta_{spat}$	Learnable parameter sets of the LSTM and GAT branches
$\alpha_{ij},N_{i}$	Graph-attention coefficient from node *j* to node *i* and neighborhood of node *i*
$W,\vec{a},h_{i}^{\prime }$	GAT transform matrix, attention vector, and updated node representation
*d*	Shared latent dimension used before multimodal fusion
$h_{temp}^{\prime },h_{spat}^{\prime }$	Projected temporal and spatial embeddings in shared latent space
$W_{t},b_{t}$ $W_{s},b_{s}$	Learnable projection parameters for temporal/spatial branches
$g_{gate},W_{1},b_{1}$ $w_{2},b_{2}$	Sample-wise modality weight and parameters of the two-layer gate
*z*	Joint spatio-temporal fused embedding
$f_{LSTM}\left(\cdot \right),f_{GAT}\left(\cdot \right)$	Temporal encoder, spatial encoder,
$f_{\mathrm{fusion}}\left(\cdot \right),g\left(\cdot \right)$	fusion, and classification mappings
σ(·)	Activation function (sigmoid where specified)
$y,\hat{y}$ $y_{i},{\hat{y}}_{i}$	Ground-truth labels and predicted anomaly probabilities (global and sample-level forms)
L,M	Loss function and number of samples used to compute it
$w_{pos}$	Positive-class weight in the WBCE loss
Φ	Overall mapping function for anomaly detection
${\hat{p}}_{i,f,s},{\hat{c}}_{i,f,s}$ $t_{f,s}^{val}$	Predicted probability, binary decision, and validation threshold for window *i*, fold *f*, and seed *s*
$\bar{d},S_{d}^{2},n$	Mean paired F1 difference, variance of paired differences, and number of paired runs
$n_{test},n_{train}$	Numbers of test and training trips used in the corrected variance
π,r,q	Anomaly prevalence, true-positive rate, and false-positive rate
κ	Sampled traffic-intensity level

**Table 3:** LSTM branch configuration.

Component	Setting	Role/Output
Input window	τ=50, 4 features	Tensor shape [B,50,4]
Input features	$v_{t}$, $a_{t}$, $x_{sin}$, $x_{cos}$	Driving-profile representation
Stacked LSTM	input size =4, hidden size =128	Temporal dependency learning
Number of layers	2	Hierarchical sequence encoding
Dropout	p=0.2	Regularization between LSTM layers
Temporal embedding	$h_{temp}\in R^{128}$	Output tensor [B,128]
Parameters	200,704	90.9% of the weighted-sum model

**Table 4:** GAT branch configuration.

Component	Setting	Role/Output
Graph input	4678 nodes, 53,660 edges	Trip-specific segment instances
Distinct physical roads	628 unique osm_segment_id	Merged-graph nodes
Node features	17 dimensions	Structural and contextual descriptors
GAT layer 1	GATConv(17, 32, heads = 2)	Intermediate tensor [N,64]
GAT layer 2	GATConv(64, 64, heads = 1)	Spatial embedding tensor [N,64]
Spatial embedding	$h_{spat}\in R^{64}$	Output of active road segment
Parameters	5568	2.5% of the full model

**Table 5:** Fusion operators and parameter counts.

Component	Setting	Fusion Parameters
Temporal projection	Linear 128→64 + ReLU	—
Spatial projection	Linear 64→64 + ReLU	—
Sample-wise gate	Linear 128→32 + tanh, 32→1 + sigmoid	4161
Fixed concatenation	$\left[h_{temp}^{\prime }∥h_{spat}^{\prime }\right]$	0
Static weighted sum	single learnable scalar *s*	1
Cross-attention	4-head, d=64	16,640
Classifier	MLP 64/128→32→1	—

**Table 6:** Road-attribute imputation defaults.

Road Type	Default Lanes	Default Speed (km/h)
motorway	3	130
motorway_link	1	80
trunk	2	110
trunk_link	1	60
primary	2	90
primary_link	1	50
secondary	1	80
secondary_link	1	40
(default)	–	50

**Table 7:** Anomaly taxonomy before and after cleaning.

No.	Subtype	Generation Condition	Generated	Cleaned
1	Speed-limit violation	$v_{t}>1.10v_{\max}$	1405	1353
2	Abnormal dwell	Stationary for >30 s	11,610	11,610
3	Abnormal acceleration	$a_{t}\in \left[-6,-3\right]\cup \left[1.5,2.5\right]$ m/s^2^	299	287
4	Accident	Composite perturbation and prolonged stop	5741	5740
5	Abnormal heading change	$|\Delta \theta_{t}|>10^{{}^{\circ}}$	213	208
6	GPS speed artifact	Perturbation of kinematic history	66	11
7	GPS acceleration artifact	Perturbation of kinematic history	76	75
8	GPS heading artifact	Perturbation of kinematic history	94	93
9	Signal freeze	Identical values for ≥2 s	606	593

**Table 8:** Fixed-partition trip and window distribution.

Partition	Trips	Sequences	Anomaly Ratio
Train	63	19,255	7.90%
Validation	13	4036	7.58%
Test	14	4799	3.27%

**Table 9:** Fixed-partition rule-based and classical baselines.

Model	Precision	Recall	F1-Score	ROC-AUC
Rule: v<1 only (0 params)	—	—	0.8129	—
Rule: v<1∨|a|>3 (0 params)	—	—	0.8317	—
Rule: 3 thresholds (0 params)	0.8354	0.8726	0.8536	—
XGBoost (window aggregates)	0.9200	0.8166	0.8651±0.0052	0.9966
LightGBM (window aggregates)	0.9355	0.8127	0.8698±0.0075	0.9966
Random Forest (window aggr.)	0.9518	0.8013	0.8699±0.0061	0.9964
LSTM only (no spatial branch)	0.9400	0.7694	0.8455±0.0190	0.9294
LSTM–GAT (weighted sum)	0.9336	0.8178	0.8713±0.0188	0.9638

**Table 10:** Fixed-partition encoder comparison.

Config	Origin	F1-Score	ROC-AUC	Params	*p* (Window)
LSTM + GAT	this study	0.8713±0.0188	0.9638	220,802	—
GRU	DCRNN backbone	0.8701±0.0102	0.9929	170,626	0.119
Transformer	Transformer	0.8698±0.0081	0.9899	292,098	0.815
Dilated causal conv	Graph WaveNet	0.8535±0.0114	0.9799	416,258	0.115
No spatial branch	control	0.8455±0.0190	0.9294	215,234	0.549
Gated TCN	STGCN	0.8314±0.0058	0.9861	122,242	0.003
Chebyshev conv	STGCN	0.8234±0.0389	0.9481	223,106	0.007
Diffusion conv	DCRNN	0.8181±0.0450	0.9633	228,290	0.003
Adaptive adjacency	Graph WaveNet	0.4657±0.2833	0.7891	321,850	<0.001

**Table 11:** Fixed-partition fusion comparison.

Fusion	F1-Score	ROC-AUC	Worst Seed	Params
Static weighted sum	0.8713±0.0188	0.9638	0.8491	1
Cross-attention	0.8559±0.0228	0.9417	0.8369	16,640
Fixed concatenation	0.8442±0.0091	0.8993	0.8339	0
Sample-wise gate	0.6953±0.3488	0.8752	0.0715	4161

**Table 12:** Primary cross-validated fusion performance.

Model	Run F1	Worst	Collapse	Pooled F1	Brier	ECE
LSTM only	0.8840±0.0380	0.8302	0/25	0.8901	0.0208	0.0548
Weighted-sum hybrid	0.8839±0.0415	0.8062	0/25	0.8891	0.0236	0.0409
Concatenation hybrid	0.8797±0.0441	0.7621	0/25	0.8528	0.0254	0.0560
Gated hybrid	0.8589±0.1458	0.1875	1/25	0.8849	0.0257	0.0579
Cross-attention hybrid	0.8558±0.1624	0.1010	1/25	0.8838	0.0267	0.0627

**Table 13:** Fusion stability across partitions and graphs.

Configuration	Trip-Instance Graph		Merged Graph	
	F1	Degenerate	F1	Degenerate
Gated	0.8612±0.1261	1/50	0.8342±0.1708	3/50
Weighted sum	0.8845±0.0443	0/50	0.8812±0.0474	0/50
Concatenation	0.8715±0.0670	0/50	0.8811±0.0400	0/50
Cross-attention	0.8304±0.1986	3/50	0.8510±0.1535	1/50
LSTM only	0.8796±0.0386	0/50	0.8796±0.0386	0/50

**Table 14:** Anomaly-subtype detection performance.

Subtype	Windows	Trips	P	R	Hybrid F1	LSTM F1	Δ	Holm *p*
Abnormal dwell	1154	33	0.9250	0.9515	0.9381	0.9307	+0.0074	1.000
Accident	569	7	0.8578	0.9438	0.8987	0.8935	+0.0052	1.000
Speed-limit violation	144	28	0.1759	0.1319	0.1508	0.2288	−0.0780	1.000
Signal freeze	62	24	0.0220	0.0323	0.0261	0.0848	−0.0587	0.172
Heading change	21	13	0.0220	0.0952	0.0357	0.0336	+0.0021	—
Acceleration	19	15	0.0111	0.0526	0.0183	0.0342	−0.0158	—
GPS speed artifact	0	0	—	—	—	—	—	—
GPS acceleration	8	8	0.0111	0.1250	0.0204	0.0377	−0.0173	—
GPS heading	8	6	0.0000	0.0000	0.0000	0.0000	0.0000	—

**Table 15:** Contextual-feature and edge controls.

Condition	F1	Δ	95% CI	Holm *p*
Real features and edges	0.8839±0.0415	—	—	—
Self-loops only	0.8878±0.0417	+0.0039	[−0.0277,+0.0354]	1.000
Shuffled feature rows	0.8926±0.0352	+0.0087	[−0.0185,+0.0359]	1.000
Random matched-density edges	0.8847±0.0399	+0.0008	[−0.0312,+0.0328]	1.000

**Table 16:** Classical baselines under grouped cross-validation.

Model	Run F1	Pooled F1	Δ vs. Gate	Trip Holm *p*
Gated hybrid	0.8589±0.1458	0.8849	—	—
LightGBM	0.9195±0.0307	0.9258	+0.0409	0.0002
XGBoost	0.9095±0.0327	0.9156	+0.0307	0.0002
Random Forest	0.9082±0.0299	0.9106	+0.0257	0.0082
Three-threshold rule	0.8938±0.0375	0.9020	+0.0171	0.2288

**Table 17:** Replication on synthetic road-network datasets.

Dataset	Best Mean F1	Significant	Equivalent	Undetermined
Kocaeli, dictionary imputation	0.9398	0/10	10/10	0/10
Kocaeli, KNN imputation	0.9247	0/10	6/10	4/10
Hamburg, dictionary imputation	0.8334	0/10	0/10	10/10
Hamburg, KNN imputation	0.8841	0/10	2/10	8/10

**Table 18:** Per-trip F1 differences and effect sizes.

Comparison	Mean Δ	SD	$d_{z}$	Favor First/Second
Weighted sum vs. LSTM	−0.0092	0.0885	−0.10	18/14
Gate vs. LSTM	−0.0146	0.0899	−0.16	15/16
Gate vs. weighted sum	−0.0054	0.0330	−0.16	12/18

**Table 19:** Fixed-partition graph comparison.

Configuration	Fusion	Nodes	Deg.	F1-Score	ROC-AUC
GAT only, trip-instance	—	4678	11.47	0.0767±0.0035	0.5910
GAT only, merged	—	628	1.11	0.0745±0.0047	0.7115
Hybrid, trip-instance	weighted sum	4678	11.47	0.8713±0.0188	0.9638
Hybrid, merged	weighted sum	628	1.11	0.8413±0.0010	0.9228
Hybrid, trip-instance	gated	4678	11.47	0.6953±0.3488	0.8752
Hybrid, merged	gated	628	1.11	0.8493±0.0274	0.9684

**Table 20:** Window-level and trip-level inference.

Family	Comparison	*p* (Window)	*p* (Trip)	Holm	Widen.
Baselines	vs. LSTM only	0.549	0.749	1.000	2.43×
	vs. LightGBM	0.302	0.219	0.657	1.81×
	vs. XGBoost	0.049	0.064	0.320	1.80×
	vs. Random Forest	0.754	0.632	1.000	1.07×
	vs. dwell-only rule	<0.001	0.065	0.320	1.84×
Fusion	gated vs. weighted sum	0.031	0.032	0.097	1.25×
	gated vs. concatenation	1.000	0.500	0.755	1.16×
	gated vs. cross-attention	0.727	0.377	0.755	0.85×
Encoders	vs. adaptive adjacency	<0.001	0.007	0.056	2.71×
	vs. gated TCN	0.003	0.048	0.335	1.44×
	vs. Chebyshev conv	0.007	0.061	0.365	2.79×
	vs. diffusion conv	0.003	0.062	0.365	2.22×
	vs. GRU	0.119	0.375	1.000	1.71×
	vs. dilated conv	0.115	0.503	1.000	1.19×
	vs. Transformer	0.815	0.439	1.000	1.17×
	vs. no spatial branch	0.549	0.748	1.000	2.43×

**Table 21:** Model size and inference latency.

Model	Params	GPU	GPU Cached	CPU	CPU Cached
Hybrid (weighted sum, GAT)	220,802	2.87 ms	0.64 ms	27.82 ms	7.03 ms
Hybrid (gated, GAT)	224,962	2.80 ms	0.78 ms	25.07 ms	7.63 ms
Hybrid (concat)	222,849	2.56 ms	0.55 ms	23.14 ms	7.32 ms
Hybrid (cross-attention)	237,441	2.91 ms	0.96 ms	19.83 ms	6.61 ms
LSTM only	215,234	0.80 ms	0.64 ms	7.69 ms	7.03 ms
GRU + GAT	170,626	2.60 ms	0.66 ms	33.63 ms	8.58 ms
Gated TCN + GAT	122,242	2.76 ms	0.88 ms	24.79 ms	1.83 ms
Dilated conv + GAT	416,258	4.22 ms	2.26 ms	27.42 ms	5.98 ms
Transformer + GAT	292,098	3.30 ms	1.30 ms	22.48 ms	2.84 ms
Adaptive adjacency + LSTM	321,850	6.18 ms	0.69 ms	55.40 ms	6.85 ms

**Table 22:** Fusion parameter counts and training cost.

Operator	Fusion Params.	Total Params.	Epochs	Minutes
Weighted sum	1	220,802	34.4	2.72
Concatenation	0	222,849	19.8	1.50
Sample-wise gate	4161	224,962	31.4	2.66
Cross-attention	16,640	237,441	30.0	2.49

## Data Availability

Synthetic data and the associated preprocessing, modeling, and evaluation code are available at https://github.com/keremkartal/Anomaly-Detection (accessed on 10 October 2026). The version archive is identified by doi:10.5281/zenodo.22637361 (v2.0.0), and the concept record by doi:10.5281/zenodo.22637360. Experimental configurations are tracked using protocol hashes, and shared configurations reuse cached runs.

## Appendix A. Selected-Study Comparison Details

**Table A1:** Detailed evaluation protocols and principal findings for the selected studies.

Study	Context and Design	Inference and Findings
(a) Evaluation procedures		
Huang et al. [4]	Partitioning: Predefined train/test; no CVRepetitions: Five runs; mean only; seeds NR	Unit and dependence: Adjustment NR (timestamp; point-adjusted scoring)Uncertainty and tests: CI and tests NR
Duan et al. [5]	Partitioning: Random 80:20 split; no CVRepetitions: Seeds NR; 1000 simulation trials averaged	Unit and dependence: Adjustment NR (10-step window)Uncertainty and tests: CI and tests NR; significance described qualitatively
Nguyen et al. [6]	Partitioning: 70/10/20 split ^a^; no CVRepetitions: Five runs for proposed model only; seeds NR	Unit and dependence: Adjustment NR (window, node, horizon)Uncertainty and tests: SD for proposed model only; paired CI NR
Liang et al. [7]	Partitioning: Partition NR; CV for teacher only; folds NRRepetitions: NR	Unit and dependence: Adjustment NR (record)Uncertainty and tests: *t*-test, p<0.05; test details NR
Cui et al. [8]	Partitioning: 7:1:2 split; test-scene vehicles excluded; no CVRepetitions: NR; single performance values	Unit and dependence: Adjustment NR (8-s segment)Uncertainty and tests: Wilcoxon test; pairing unit NR
This study	Partitioning: Trip-grouped 70/15/15 split; five-fold CV; five repeated grouped partitionsRepetitions: Seeds 42–46 for fixed split and primary CV; two shared seeds for repeated CV	Unit and dependence: Trip clusters for 80%-overlapping windows; approximate correction for shared training setsUncertainty and tests: Trip-bootstrap CI; trip-level permutation; corrected resampled tests; Holm; equivalence tests
(b) Task context and principal results		
Study	Task and data	Reported claim or empirical finding
Huang et al.	Multivariate time-series anomaly detection; SWaT, SMAP, MSL, SMD	Approximately two percentage points higher F1 than the strongest baseline
Duan et al.	Highway anomaly detection; METR-LA, PEMS-BAY	F1 0.967 versus 0.945 for static detection
Nguyen et al.	Traffic-volume and speed forecasting; Dublin2021 and six further datasets	Up to 12.3% lower mean absolute error
Liang et al.	Network intrusion detection; BoT-IoT, TON_IoT, UNSW-NB15	99.28% accuracy on UNSW-NB15
Cui et al.	Trajectory prediction; NGSIM, HighD	Root mean squared error reduced by 0.27–0.80 m on NGSIM
This study	Traffic anomaly detection; 90 synthetic trips; primary CV: 25 runs per configuration; repeated CV: 50 runs per graph–configuration cell	Fusion rankings change with seed coverage; repeated-partition inference establishes neither pairwise superiority nor equivalence

Notes: NR: not reported; CV: cross-validation; SD: standard deviation; CI: confidence interval. NR indicates a reporting gap and does not establish that a procedure was absent. ^a^ The 70/10/20 split is specified for Dublin2021; partition details for the other datasets are NR. Results across these tasks are not directly comparable.

## Appendix B. Generation Diagnostics and Operator Settings

### Appendix B.1. Empirical Traffic-State Transitions

Table A2 reports first-order transitions between consecutive non-anomalous records within each trip. States use a stop threshold of 1 km/h and an acceleration dead band of 0.10 m/s^2^. Entries are empirical frequencies, and occupancy is the fraction of records in each state; these estimates describe realized behavior rather than simulator transition parameters.

**Table A2:** Empirical traffic-state transitions.

Regime	From	Stop	Decel.	Const.	Accel.	Occupancy
Free flow	Stop	0.0000	0.0000	0.0000	1.0000	0.0001
	Decel.	0.0000	0.0290	0.0027	0.9683	0.3367
	Const.	0.0000	0.0761	0.6448	0.2791	0.0126
	Accel.	0.0000	0.5022	0.0058	0.4921	0.6507
Transition	Stop	1.0000	0.0000	0.0000	0.0000	0.0029
	Decel.	0.0000	0.047	0.002	0.951	0.3283
	Const.	0.0000	0.3333	0.4676	0.1991	0.0045
	Accel.	0.0000	0.460	0.003	0.537	0.6643
Congested	Stop	0.9613	0.0000	0.0000	0.0387	0.0565
	Decel.	0.008	0.171	0.001	0.819	0.3518
	Const.	0.0000	0.6637	0.2035	0.1327	0.0039
	Accel.	0.0000	0.4732	0.0048	0.5220	0.5878

### Appendix B.2. Encoder and Fusion Settings

Table A3 lists the task-specific operator settings. Parameters use PyTorch and PyTorch Geometric default initialization under the stated training seeds. The configurations do not result from exhaustive hyperparameter searches.

**Table A3:** Encoder and fusion operator settings.

Component	Setting	Value
LSTM	Hidden size; layers; dropout	128; 2; 0.2
GRU	Hidden size; layers; dropout	128; 2; 0.2
STGCN temporal adaptation	Kernel size; blocks	3; 2
Graph WaveNet temporal adaptation	Kernel; layers; dilations	2; 4; 1–8
Transformer	Heads; layers; feed-forward width	4; 2; 256
GAT	First-layer width; output; heads	32; 64; 2
STGCN spatial adaptation	Chebyshev order *K*	3
DCRNN spatial adaptation	Diffusion steps *K*	2
Graph WaveNet spatial adaptation	Node-embedding width; *K*	10; 2
Branch projections	Output width	64
Classifier	Hidden width	32
Sample-wise gate	Widths; hidden activation	128–32–1; tanh

### Appendix B.3. Learning-Rate Selection

Learning rates are selected by validation F1 from $\left\{5\times 10^{-4},10^{-3},2\times 10^{-3}\right\}$. Table A4 reports fixed-test F1 as the mean ± standard deviation over five seeds at the selected rate. The epoch column gives the mean selected checkpoint epoch, distinct from the total training duration.

**Table A4:** Validation-selected learning rates and checkpoint epochs.

Configuration	Learning Rate	F1	Epoch
LSTM–GAT	$10^{-3}$	0.8713±0.0188	19.4
GRU temporal	$10^{-3}$	0.8701±0.0102	25.6
Transformer temporal	$10^{-3}$	0.8698±0.0081	23.2
Graph WaveNet temporal	$10^{-3}$	0.8535±0.0114	21.0
STGCN temporal	$10^{-3}$	0.8314±0.0058	14.0
Chebyshev spatial	$10^{-3}$	0.8234±0.0389	24.0
Diffusion spatial	$10^{-3}$	0.8181±0.0450	30.4
Adaptive adjacency	$5\times 10^{-4}$	0.8113±0.0719	8.2

## Appendix C. Detailed Sensitivity Results

### Appendix C.1. Window Length and Stride

Table A5 counts out-of-fold windows across all 90 trips. Each condition uses five folds and one training seed; mean F1 and standard deviation summarize the five fold-specific scores. The supplementary reference comes from separate sensitivity runs rather than the cached primary runs in Table 12. Paired comparisons use corrected run-level inference. The seed identifier is unspecified, and the reported *p*-values are not identified as Holm-adjusted.

**Table A5:** Window-length and stride sensitivity.

τ	Stride	Overlap	Windows	Hybrid/LSTM F1	Δ	*p*
25	10	60%	28,321	0.8990±0.0384/0.8721±0.0674	+0.0269	0.537
25	25	0%	11,350	0.7826±0.2188/0.8694±0.0460	−0.0868	0.579
50	10	80%	28,090	0.8865±0.0360/0.8871±0.0468	−0.0006	0.981
50	25	50%	11,260	0.8519±0.0485/0.8591±0.0529	−0.0072	0.647
50	50	0%	5654	0.7957±0.0694/0.8441±0.0569	−0.0483	0.354
100	10	90%	27,640	0.8706±0.0536/0.9066±0.0400	−0.0360	0.321
100	25	75%	11,080	0.8424±0.0599/0.8625±0.0553	−0.0201	0.694
100	50	50%	5564	0.6246±0.3594/0.6984±0.2448	−0.0739	0.846

### Appendix C.2. Anomaly Prevalence

Table A6 reweights class prevalence while retaining the class-conditional detection rates from five folds and two seeds. The values are analytical evaluations of F1 under fixed rates, rather than results from independently regenerated datasets.

**Table A6:** F1 sensitivity to anomaly prevalence.

Configuration	1%	3%	8%	15%	25%
Gated hybrid	0.759	0.857	0.895	0.907	0.912
Weighted-sum hybrid	0.730	0.837	0.885	0.900	0.907
LSTM only	0.775	0.858	0.889	0.898	0.903

### Appendix C.3. Added Speed Noise

Table A7 reports descriptive weighted-sum F1 under inference-time speed perturbations with fixed decision thresholds. Noise magnitude σ is expressed in km/h, and the AR(1) process has ρ=0.9. Acceleration is recomputed from perturbed one-second speed differences, preserving its relationship to the speed channel. The number and identifiers of the fitted models used in this experiment remain unspecified.

**Table A7:** F1 sensitivity to added speed noise.

Noise	σ=0	0.5	1	2	5	10
White	0.904	0.902	0.899	0.783	0.145	0.034
AR(1)	0.904	0.902	0.900	0.890	0.767	0.448

### Appendix C.4. Imputed Road Attributes

The controls in Table A8 are retrained over five folds and five seeds (25 runs per condition), retaining the original trajectories and labels. F1 differences are paired against the released-feature reference, and confidence intervals use the corrected resampled variance in Equation (22).

**Table A8:** Road-attribute imputation sensitivity.

Treatment	Features	F1	Δ	95% CI
As released	17	0.8839±0.0415	—	—
Imputation indicators added	19	0.8755±0.0634	−0.0084	[−0.0719,+0.0551]
Road-type median filling	17	0.8821±0.0484	−0.0018	[−0.0497,+0.0460]
Speed limit and lanes removed	15	0.8774±0.0436	−0.0065	[−0.0414,+0.0284]
